# Mortality Advantage Reversed: The Causes of Death Driving All-Cause Mortality Differentials Between Immigrants, the Descendants of Immigrants and Ancestral Natives in Sweden, 1997–2016

**DOI:** 10.1007/s10680-022-09637-0

**Published:** 2022-10-27

**Authors:** Matthew Wallace

**Affiliations:** Sociology Department, Stockholm University Demography Unit, Stockholm, Sweden

**Keywords:** Health, Mortality, Causes of death, Immigrants, Descendants, Integration

## Abstract

A small but growing body of studies have documented the alarming mortality situation of adult descendants of migrants in a number of European countries. Nearly all of them have focused on all-cause mortality to reveal these important health inequalities. This paper takes advantage of the Swedish population registers to study all-cause and cause-specific mortality among men and women aged 15–44 in Sweden from 1997 to 2016 to a level of granularity unparalleled elsewhere. It adopts a multi-generation, multi-origin and multi-cause-of-death approach. Using extended, competing-risks survival models, it aims to show (1) how the all-cause mortality of immigrants arriving as adults (the **G1**), immigrants arriving as children (the **G1.5**) and children of immigrants born in Sweden to at least one immigrant parent (the **G2**) differs versus ancestral Swedes and (2) what causes-of-deaths drive these differentials. For all-cause mortality, most **G1** (not Finns or Sub-Saharan Africans) have a mortality advantage. This contrasts with a near systematic reversal in the mortality of the **G1.5** and **G2** (notably among men), which is driven by excess *accident and injury*, *suicide*, *substance use* and *other external cause* mortality. Given that external causes-of-death are preventable and avoidable, the findings raise questions about integration processes, the levels of inequality immigrant populations are exposed to in Sweden and ultimately, whether the legacy of immigration has been positive. Strengths of the study include the use of quality data and advanced methods, the granularity of the estimates, and the provision of evidence that highlights the precarious mortality situation of the seldom-studied **G1.5**.

**Supplementary Information:**

The online version contains supplementary material available at 10.1007/s10680-022-09637-0.

## Introduction

The descendants of immigrants represent one of the fastest growing and diverse parts of young populations in a number of high and middle-income countries (Suárez-Orozco, 2018). In Europe, the second-generation (**G2**, those with parents who migrated before they were born) (Lessard-Phillips et al., 2015) comprise 6% of the total population; shares are even higher in countries such as France (14.3%), Sweden (11.2%) and Belgium (11.0%) (Agafiţei & Ivan, 2016). The descendants of immigrants can also be immigrants themselves—having moved with their parents prior to becoming adults (**G1.5**) (Lessard-Phillips et al., 2015). Statistics on the **G1.5** are scarcely recorded; they are often combined with immigrants arriving as adults (**G1**). Yet, they are a distinct group, representing a bridge between the **G1** and **G2** in that they move—like the **G1**—but spend (at least some of) their youth in the host country—like the **G2** (Mehta et al., 2019).

An emerging body of research has highlighted the concerning mortality situation of the **G2** (De Grande et al., 2014; Guillot et al., 2019; Khlat et al., 2019; Manhica et al., 2015; Mehta et al., 2019; Singh & Siahpush, 2001; Tarnutzer et al., 2012; Vandenheede et al., 2014, 2015; Wallace, 2016) and the **G1.5** (De Grande et al., 2014; Mehta et al., 2019). This is alarming because these mortality *disadvantages* contrast with the sizeable mortality *advantage*s of the **G1** (Aldridge et al., 2018) and because this reversal seemingly occurs within a single generation. This leads to questions surrounding the success of integration processes, the inequality faced by immigrants after arriving in the new country and, ultimately, whether the legacy of migration—best captured through the lives of their descendants—has been positive (Portes & Rumbaut, 2001).

Most studies have focused on all-cause mortality to reveal these important health inequalities. Although existing studies *have* examined cause-specific mortality, they have typically analysed a single cause-of-death, studied very broad cause-of-death categories (e.g. natural and external causes) (Manhica et al., 2015; Mehta et al., 2019), or investigated detailed causes-of-death for one specific origin (e.g. Hispanics in the U.S.) (Eschbach et al., 2007). This article expands the evidence base by adopting an extended, competing-risks survival analysis approach. The value of this approach lies in its ability to show how all-cause and cause-specific mortality vary among the **G1**, **G1.5** and **G2** relative to a given reference population—as in existing studies—and, *crucially*, reveal how the variation in each cause-of-death combines to generate the observed all-cause mortality differences—something that has not been seen in this literature before. The article also offers evidence to a level of detail unparalleled elsewhere, using a multi-generation, multi-origin approach to study mortality among men and women from eight specific causes-of-death.

Beyond the value of the approach and granularity of the estimates, studying cause-of-death has both theoretical and practical value. Explanations of the **G1** mortality advantage and its reversal among the **G1.5** and **G2**—the *healthy migrant effect*, *cultural factors*, *social conditions*, *psychosocial factors* and *data-driven explanations* (Wallace, 2016)—have predominantly been developed using estimates of all-cause mortality. Yet, cause-specific mortality might be expected to vary in distinct ways across generations according to these different explanations. Thus, the provision of estimates by cause-of-death can provide more nuanced evidence against which to assess their viability. The study will also show whether the overall disadvantages of the **G1.5** and **G2** reflect systematic excesses across various causes-of-death or elevated mortality in a limited number of causes-of-death. Such information has public health value, especially if the causes in question are *preventable* (via effective public health and primary interventions), *treatable* (avoided through timely and effective health care intervention, including secondary prevention and treatment) and, ultimately, *avoidable* (OECD, 2019)*.*

The study uses total population register data from Sweden to examine mortality among people aged 15 to 44 between 1997 and 2016. It has two aims. **First**, to document all-cause mortality differences between the **G1**, **G1.5** and **G2** relative to the ancestral Swedish population. **Second**, to identify the cause (or causes) of death driving these differences. Three research questions are defined:

1. *How does all-cause mortality vary among the ****G1****, ****G1.5****, and ****G2**** relative to ancestral Swedes, including by origins and sex?*
2. *How does mortality from specific causes-of-death vary among the ****G1****, ****G1.5****, and ****G2**** relative to ancestral Swedes, including by origins and sex?*
3. *How does the variation in cause-specific mortality combine to produce the all-cause mortality differentials observed among the ****G1****, ****G1.5****, and ****G2**** relative to ancestral Swedes?*

Aside from being home to some of the highest quality and most complete data in the world (Maret-Ouda et al., 2017), Sweden represents a fascinating context. It is considered one of the most “migrant-friendly” European Union (EU) states and one of its most diverse societies (Schierup & Ålund, 2011). It has one of the highest shares of **G1** (19.6%) and **G2** (11.2%) of any EU state (Agafiţei & Ivan, 2016). Many of the first-generation (three in four) were born outside of the EU (Agafiţei & Ivan, 2016), owing to Sweden’s liberal refugee policy (Karlsdottir et al., 2018). Yet, while Sweden is often viewed as a country with a strong welfare system and low levels of inequality, its social inequality gaps are among some of the fastest growing in Europe, notably in people with a migrant background (Trygged & Righard, 2019). Its health inequality gaps are also large compared to countries that have less developed welfare systems (Mackenbach et al., 2016).

## Background

### Pervasive Adult “Migrant Mortality Advantage”

Adult mortality among the descendants of immigrants is typically studied with reference to the “migrant mortality advantage”, which refers to the low mortality of immigrants relative to the native-born population of the host country (Guillot et al., 2018). In the past several decades, it has been pervasively documented, with systematic reviews and meta-analyses finding globally low immigrant mortality (Aldridge et al., 2018; Shor & Roelfs, 2021). Nonetheless, this migrant mortality advantage is far from universal. Its presence and scale can vary according to factors like age, country of birth, age at arrival, length of stay, and reason for arrival (Chiswick et al., 2008).

### Elevated Mortality Among the Descendants of Immigrants

Increasingly, the evidence suggests that the retention of the “migrant mortality advantage” among the descendants of immigrants is rare. In the USA, it is more common to observe a partial or complete attenuation across generations from lower immigrant mortality towards the higher mortality of the non-Hispanic White population (Singh & Siahpush, 2001, 2002). In Europe, on the other hand, a reversal across generations above the mortality of ancestral native-born is much more common (Bennet et al., 2020; De Grande et al., 2014; Guillot et al., 2019; Khlat et al., 2019; Manhica et al., 2015; Tarnutzer et al., 2012; Vandenheede et al., 2014, 2015; Wallace, 2016).

In general, excess mortality is more frequent among those descendants with parents from non-Western countries. For example, among **G2** Northern Africans—but not Southern Europeans—in France (Guillot et al., 2019), **G1.5** and **G2** Turks, Moroccans and Sub-Saharan Africans—but not Italians or Germans—in Belgium (De Grande et al., 2014; Vandenheede et al., 2014, 2015), and **G2** Middle Easterns and other non-Europeans—but not other Western countries or Eastern Europe—in Sweden (Manhica et al., 2015). Exceptions exist in the elevated mortality of **G2** Finns in Sweden (Manhica et al., 2015), Irish in the UK (Harding et al., 1996) and French in Belgium (Vandenheede et al., 2015). However, for these specific origins there is no mortality advantage to retain in the first place—their respective **G1** groups also have elevated mortality (Vandenheede et al., 2015; Wallace & Kulu, 2015; Wallace & Wilson, 2020). Moreover, prior studies *have* documented lower mortality among the descendants of non-Western immigrants, notably among **G2** Hispanics and Asian & Pacific Islanders in the US (Singh & Siahpush, 2001, 2002).

Additionally, the presence and size of the relative excess mortality is typically stronger among male descendants of immigrants. For example, in the elevated mortality found among male—but not female—**G2** Northern Africans in France (Guillot et al., 2019; Khlat et al., 2019), **G2** Italians in Switzerland (Tarnutzer et al., 2012), and **G2** Finns, former Yugoslavians, Middle Easterns and other non-Europeans in Sweden (Manhica et al., 2015). As above, exceptions exist in the greater excess among **G2** Sub-Saharan African women in Belgium (Vandenheede et al., 2015).

Moving beyond all-cause mortality, studies consistently find that the lower cancer mortality of the **G1**—with differences by cancer site—almost entirely disappears among the **G2** (Balzi et al., 1995; Bennet et al., 2020; Eschbach et al., 2007; Hemelrijck et al., 2017; Hemminki & Li, 2002a, 2002b; Hemminki et al., 2002; Parkin & Iscovich, 1997; Parkin & Khlat, 1996; Thomas & Karagas, 1987). Circulatory disease mortality has also been found to attenuate over generations, including among Hispanics in the US (Eschbach et al., 2007), and Italians, French, Moroccans, Turks, and Sub-Saharan Africans in Belgium (Vandenheede et al., 2014, 2015). Yet, some studies find elevated coronary heart disease mortality in both the **G1** *and* **G2** (e.g. among Finns, Central & Eastern Europeans and Turks in Sweden) (Sundquist & Li, 2006). Two studies from Sweden observe elevated mortality from *all* external causes among the **G1.5** and **G2** (Manhica et al., 2015; Mehta et al., 2019). Eschbach et al. (2007) document higher homicide, substance use, and other accident & injury mortality among **G2** Hispanics compared to **G1**, but only homicide mortality is higher than the level of Non-Hispanic Whites. The **G2** consistently have higher suicide mortality than the **G1**, with mortality levels that are closer to—or even above—the levels of their respective ancestral native-born populations (Bauwelinck et al., 2017; Dunlavy et al., 2018; Hjern & Allebeck, 2002; Thiene et al., 2015).

### Mechanisms

People who move between countries may be selected *directly* upon their good health and *indirectly* upon factors associated with good health (Guillot et al., 2018). The same individuals may also be selected based upon personality traits such as resolve, resilience and risk averseness (Boneva & Frieze, 2001). For the **G1**, this selection might be expected to result in lower mortality across causes-of-death, due to in-selection forces generating lower mortality from causes related directly to health and the presence of personality traits leading to lower mortality from external causes-of-death. For the **G1.5**, selection is said to be weaker or non-existent because they play no role in the decision to migrate (Guillot et al., 2018). For the **G2**, selection should play no role in their mortality.

Some **G1** origin groups may practice healthier behaviours than the normative behaviours of the host country due to the cultural norms associated with their origin country (Guillot et al., 2018). These behaviours, which may include smoking, drinking and diet, would generate lower overall mortality *and* lower mortality from diseases & medical conditions for which these behaviours are risk factors. For some origins, cultural and religious attitudes toward drug use and suicide may result in lower mortality from these external causes-of-death. The effect of cultural factors among the **G1.5** and **G2** would depend upon the extent of the intergenerational transmission of cultural norms (Spallek et al., 2011). If no such transmission takes place—and the **G1.5** and **G2** largely practice the normative behaviours of the host country—then their all-cause mortality—and risk for specific causes-of-death—should more closely resemble the ancestral native-born population.

While immigrants spend their formative years in their origin country, descendants spend them in the host country. There may be factors linked to this crucial formative period that *positively* affect **G1** mortality and *negatively* **G1.5** and **G2** mortality, e.g. socioeconomic position (SEP) (Spallek et al., 2011). Few, if any, studies have examined the role of childhood SEP on the *adult* mortality of the **G1.5** and **G2**. In the general literature, a link has been identified between poor childhood SEP and adult mortality. Various reviews show that risk for all-cause mortality is higher among individuals that experience adverse SEP during childhood. The pattern is valid for men and women but can vary by cause—with cardiovascular diseases, respiratory diseases and external causes linked, but certain cancers (typically non-smoking) not linked, to early life SEP (Galobardes et al., 2004, 2006, 2008; Montez & Hayward, 2011). If immigrants experience better (relative) childhood SEP conditions in the *origin* country, then they might have lower mortality from those particular causes-of-death linked to this early formative period. If their descendants experience worse childhood SEP conditions in the *host* country, then the opposite might well be true.

Inverse associations between adult SEP and mortality exist across a wide range of causes-of-death, including circulatory diseases, cancer, diabetes, chronic obstructive pulmonary disease and external causes (Rosvall et al., 2006). The contribution of causes-of-death to socioeconomic differences in all-cause mortality can vary over age, with external causes providing the greatest contributions at young adult ages—notably among men (Rosvall et al., 2006). For the **G1**, any effect of adult SEP on their mortality is complicated by the “socioeconomic paradox”, a well-known phenomenon in which immigrants have lower mortality than native-born populations despite being, on average, more socioeconomically disadvantaged than them (Khlat & Darmon, 2003). Among their descendants—for whom no such paradox exists—the risk of death from specific causes might more closely resemble these traditional patterns. Previous work finds that adult SEP—notably education level and occupation—can explain excess mortality among the descendants of immigrants in France and Belgium (De Grande et al., 2014; Khlat et al., 2019; Vandenheede et al., 2014), but not fully in Sweden or the UK (Manhica et al., 2015; Wallace, 2016).

Perhaps in part due to self-selection, immigrants have specific traits, such as being more decisive, resilient, and risk-averse than non-migrants are (Boneva & Frieze, 2001; Chiswick et al., 2008). These traits help them to cope with the physical, psychological and social challenges of immigration (e.g. racism and discrimination) (Gushulak, 2007). These are challenges that the **G1** expect and may be willing to endure in the interest of furthering their family’s future (Anson, 2004). The **G1.5** and **G2** may lack the necessary traits to overcome such challenges and/or are exposed to these challenges in childhood when they are much more susceptible to their potentially adverse effects (Hjern & Allebeck, 2002). This vulnerability may be enhanced by a change in reference group between generations—from origin to host country—leading to more negative evaluations of their situations, alongside higher expectations of their parents (Alba & Waters, 2011). Adverse psychosocial effects include stress, hostility, depression, feelings of hopelessness and adoption of riskier behaviours that include smoking and drug use (Macleod & Smith, 2003). The **G1** might experience low mortality from causes-of-death previously associated with psychosocial factors, such as smoking and external causes such as accidents & injuries, suicides and substance use, while the **G1.5** and **G2** might conversely experience an increased risk of death.

The *salmon bias effect* proposes that immigrants who are in poor health return to their country of origin. Only healthy immigrants who stay in the host country are included in the mortality estimates, which are not representative of all those **G1** that originally migrated (Guillot et al., 2018). If this out-selection effect is occurring on a large scale, this might conceivably generate lower mortality among the **G1**, when their true mortality is closer to that of the **G1.5** and **G2**. This bias might be more visible in causes-of-death that have a longer lag time between diagnosis and death, such as cancer, when the **G1** are still physically able to return to, and plan their death in, their country of origin.

Finally, some argue that the migrant mortality advantage is produced by substantial data problems, notably incorrect or missing emigration dates and/or missing death events (Guillot et al., 2018). If the **G1.5** and **G2** are less prone to these errors—and the level of the error in the **G1** is large, this might generate lower mortality among the **G1** when their true mortality is closer to that of the **G1.5** and **G2**. One might expect to document a higher mortality risk from ill-defined causes due to the increased risk of death abroad among the **G1**, combined with the low quality of reporting in some countries relative to Sweden (Brooke et al., 2017).

### The Swedish Context

Sweden is a monarchy in Northern Europe with a parliamentary form of government (Healy & McKee, 2004). It was transformed into a country of immigration due to the arrival of European refugees during World War II (Migrationsverket, 2020). Following the war, Sweden continued to receive labour immigrants—notably from Finland in the 1960s and 1970s (Migrationsverket, 2020). This was driven by agricultural decline in Finland, combined with demand for unskilled labour in Sweden (Korkiasaari & Söderling, 2003). Their migration was facilitated by the 1954 *Nordic Common Labour Market* agreement and for those Swedish-speaking Finns (a linguistic minority in Finland) —an ethno-linguistic affinity to their “mother country” (Hedberg & Kepsu, 2003). In response to rising immigration, Sweden began to develop integration policies. They were driven by the *Social Democratic welfare state regime*, a model known by its generous and redistributive benefits and universal welfare services meant for the entire resident population. Immigrants were granted unrestricted access to the welfare state—including the health care system—so as not to undermine the principles of this core universal egalitarianism (Borevi, 2014).

Following the implementation of an official “immigration stop” in 1972, the inflow of foreign workers was more or less replaced by refugees and family members (Borevi, 2014). This began with the refugee arrivals from Chile in the 1970s. Integration policy in the 1970s represented a radical shift towards affirming and supporting immigrants’ identities (through the retention of their own language, support for cultural activities, and maintenance of links with the country of origin) (Borevi, 2014). The arrival of refugees from Iran, Iraq, Lebanon, and Eritrea in the 1980s and the Balkans in the 1990s (Migrationsverket, 2020). By the late 1990s, although many of the measures introduced to help immigrants retain their identity remained intact, policy rhetoric had shifted toward no longer supporting this process in the long-term (Borevi, 2014). In 2001, Sweden’s Schengen membership resulted in an increasing number of European Union (EU) immigrants moving to Sweden for work and study (Migrationsverket, 2020). Most recently, the European immigrant crisis of the 2010s led to the large-scale arrival of Syrian refugees (Migrationsverket, 2020).

Despite Sweden’s multicultural outlook, there is nevertheless evidence of segregation in the labour market, with Nordic migrants focused in manufacturing, recycling and construction, Africans in caring professions and other non-Europeans in personal & cultural services (Englund, 2003). There is also evidence of residential segregation among non-Western immigrants (Malmberg et al., 2018). Regarding health care, Sundquist (1993) finds no difference in primary care visits between all immigrants and Swedes. However, Asian and African immigrants make fewer visits to primary care, while Latin American immigrants make more. Immigrants do have a higher hospital admission risk, notably Latin American and Nordic immigrants (Sundquist, 1993). Hjern et al. (2001) find that Chileans, Iranians and Turks are more likely to have recently consulted with a physician than Swedes, but are also more likely to report unmet needs and incontinuity in health care.

### Expectations

Based upon findings from the existing literature—particularly in Sweden—several expectations can be stated. First, **migrants will have lower all-cause mortality than ancestral Swedes**, excluding migrants from Finland, who have been repeatedly found to have elevated mortality in Sweden. Second, the **descendants of migrants (the G1.5 and G2) will have higher mortality than ancestral Swedes,** particularly men and descendants with parents from non-Western countries (as prior empirical evidence from Sweden and other European countries shows). Third, **the G1 might have systematically lower mortality across causes-of-death**, while **the G1.5 and G2 might have higher mortality across causes-of-death**, with some variation by origin and sex. Given the importance of external causes-of-death to the age range being studied, **variation in external causes-of-death relative to ancestral Swedes should play a key role in total mortality variation between the population subgroups**.

## Data and Methods

### The Swedish Registers

This study uses the collections of Swedish register data “*Ageing Well*” organised at Stockholm University. This data is accessible for research under ethical approval from the regional ethics board in Stockholm. It comprises longitudinal individual-level data from several administrative datasets. Available data covers the total population of Sweden annually from 1961 until 2020. This paper focuses on the period 1997–2016 due to the smaller number of (non-Western) adult descendants and deaths before 1997 and lack of information on cause-of-death after 2016. Data is merged over four registers: total population, migration, multigenerational and cause-of-death registers.

### Defining Immigrants and the Descendants of Immigrants

The **G1** are defined as foreign-born arriving in Sweden on or after age 15. The **G1.5** are defined as foreign-born arriving in Sweden from ages 0 to 14. The **G2** are defined as individuals born in Sweden who have *at least* one foreign-born parent. Ancestral Swedes are defined as individuals born in Sweden to two parents born in Sweden. Mortality hazard ratios are estimated at three levels (a) generational (ancestral Swedes, **G1**, **G1.5**, **G2**), (b) generation by western and non-western origins and (c) specific origins (Finland, other Nordic, other Western countries, Central & Eastern Europe, the Middle East, Central and Southern America, Sub-Saharan Africa, and Asia).

### Mortality

An indicator of all-cause mortality is derived from the death register using exact date of death. Causes of death is categorised (*ICD-10*) into cancers (**C00-D49**), circulatory diseases (**I00-I99**), other diseases & medical conditions (**A00-B99**; **D50-H99**; **J00-Q99**), accidents & injuries (**V00-V99**; **W00-W99**; **X00-X39**; **X45-X59; Y85**; **Y86**), suicide (**X60-X84**; **Y87**), substance use (**F11-F16**; **F18-F19**; **X40-X44**; **Y10-Y14**), other external causes-of-death (**X85-X99**; **Y00-Y84**; **Y88-Y99**) and ill-defined causes (**R00-R99**). Mortality is analysed between ages 15 and 44. This is because of the particularly young age structure (see supplementary Figure S1) and age-at-death distribution (see supplementary Figure S2) of **G1.5** and **G2** who have non-Western origins.

### Survival Setup and Analysis

To examine the mortality of the **G1**, **G1.5** and **G2** relative to the ancestral Swedes, all-cause and cause-specific mortality hazard ratios (HRs) are estimated using competing-risks survival analysis.

Entry into the analyses can begin in several ways. Individuals resident in Sweden aged between 15 and 44 on 1st January 1997 become “at risk” immediately. People resident in Sweden on 1st January 1997 who are younger than age 15 become “at risk upon” reaching age 15, as long as that occurs before 31st December 2016 and that person is still resident in Sweden. People who are not resident in Sweden on 1st January 1997 but assume residency on a later date—as long as that date is before 31st December 2016—become “at risk” from their date of arrival in Sweden (if aged 15 to 44). Finally, individuals arriving in Sweden between 1st January 1997 and 31st December 2016 who are younger than age 15 on their date of arrival will become “at risk” upon reaching age 15, as long as that occurs before 31st December 2016. Residency is verified at the end of each calendar year in the register data, with a variable indicating their specific county of residence.

Exit from the analysis takes place at the age-of-death (for people who die between ages 15 and 45). Otherwise, people are right-censored (a) at their age of emigration (where an emigration is registered), (b) age at the end of the year that they are no longer classed as resident in Sweden (where an emigration is not registered), at age 45 (if individuals reach age 45 alive before the end of 2016), or (c) their age at the end of 2016 if they have neither died, emigrated nor reached age 45.

An extended survival setup is implemented, whereby if there are *K* competing events (in this case eight causes-of-death categories), each person requires *K* rows in a long-form data file—one representing each potential cause of death. A column variable “cause” is used to denote the event type (cause-of-death). The value of the time variables remains identical over the *K* rows of each person, but the event variable changes. Instead of values *0, 1,…, K*, the event variable takes on the value 1 if the corresponding event type (i.e. the cause of death) is the one that occurred (and 0 if it did not). The values of covariates are replicated for individuals over the *K* rows.

Cox Proportional Hazards models are then fitted; age represents the timescale in the regression models. With the setup as described above, the assumption is made that baseline cause-specific hazards are proportional. Even though this assumption often proves to be unrealistic, this kind of proportional risk model boasts the nice property that the probability of a person failing due to cause-of-death *k* follows a logistic model (Putter et al., 2007). Robust estimates of standard errors are estimated to correct for the correlation that is caused by the multiplication of the data set.

### Modelling Strategy

**Fig. 1:**
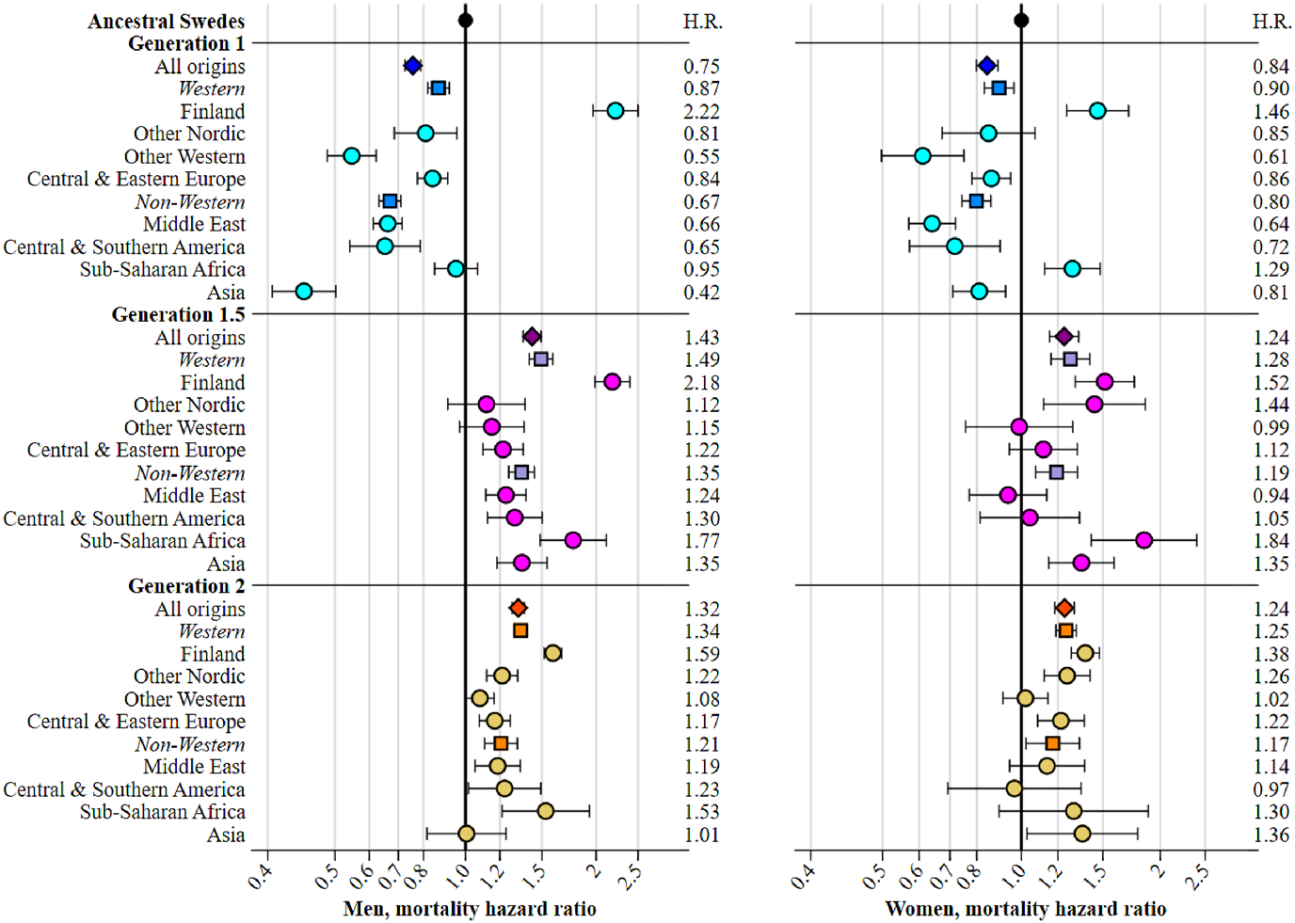
Age and birth cohort adjusted hazard ratios of all-cause mortality by sex, generation and lower level origins in Sweden, 1997–2016, ages 15–44. *Notes:* full regression models available in Table S1 (Model 1a), Table S2 (Model 1b), and Table S3 (Model 1c). Source: author’s calculation based upon the Swedish register data collection “Ageing Well”

Models 1a-c adjust for age, birth cohort, cause-of-death (with cancer as the reference), and one of (a) generation (ancestral Swedes, **G1**, **G1.5** and **G2**), (b) generation by western, non-western origin, or (c) generation by individual and parental origins. Ancestral Swedes always act as the reference group. This model reveals all-cause mortality differences between ancestral Swedes, immigrants, and descendants of immigrants at increasing levels of origin detail. Hazard ratios from the models, shown in Fig. 1, help to answer research question 1. The model is expressed as:

$$\ln \mu \left( t \right) = \ln \mu_{0} \left( t \right) + \propto k + \gamma Z + \beta x$$

whereby ${\varvec{\mu}}\left( {\varvec{t}} \right)$ represents the hazard of mortality at age${\varvec{t}}$, ${{\varvec{\mu}}}_{0}\left({\varvec{t}}\right)$ represents the baseline hazard, $\propto$ denotes the effect of the ***k*th** cause of death (***k*** = 1…8) on all-cause mortality, ${\varvec{\gamma}}$ denotes the effect of immigrant-origin population variable${\varvec{Z}}$, and ${\varvec{\beta}}$ denotes the effect of birth cohort variable${\varvec{x}}$.

Models 2a-c adjust for age, birth cohort and combinations of cause-of-death by (a) generation, (b) generation by western, non-western and (c) generation by individual and parental origins. Cancer mortality among ancestral Swedes is the reference. Hazard ratios derived from these models, shown in Tables 3 and 4, answer research questions 2 and 3. The model is specified as follows:

$$\ln \mu \left( t \right) = \ln \mu_{0} \left( t \right) + y_{k} Z + \beta x$$

whereby the immigrant status variable **Z** is now permitted to vary according to specific causes of death.

From a potentially eligible starting sample of 6,666,295 people, 99.07% of the total population of Sweden aged 15–44 between 1997 and 2016 was retained. 41,180 (0.62%) of people were dropped due to lack of information on country of birth, while 20,576 (0.32%) of people were dropped due to issues with event dates in the survival setup. This left a final analytical sample of 6,604,539 people and 43,419 deaths. 95% Confidence intervals are provided, as a measure of population variance, but the hazard ratios do represent the entire defined subpopulations of Sweden.

## Results

RQ1 “How does all-cause mortality vary among the G1, G1.5, and G2 relative to ancestral Swedes, including by origins and sex?”

Table 1 presents the frequency and percentage of risk-time and deaths, whereby risk-time refers to the time spent, in years, “at risk” of death after meeting the entry criteria described in Sect. 4.4.

**Table 1:** Risk-time (years) and deaths by sex, generation, and lowest level origins in Sweden, 1997–2016, people aged 15–44

Sex by individual or parental birth region	Generation 1				Generation 1.5				Generation 2			
	Risk-time	*%*	Deaths	*%*	Risk-time	*%*	Deaths	*%*	Risk-time	*%*	Deaths	*%*
Men												
Ancestral Swedes	26,225,000		20,270									
All regions	3,821,015	*100*	2,480	*100*	2,002,795	*100*	1,905	*100*	4,587,741	*100*	4,387	*100*
Western	1,635,236	*43*	1,253	*51*	929,166	*46*	1,029	*54*	3,735,365	*81*	3,847	*88*
Finland	118,803	*3*	272	*11*	213,850	*11*	463	*24*	1,608,038	*35*	1,983	*45*
Other Nordic	194,073	*5*	140	*6*	105,896	*5*	92	*5*	592,503	*13*	586	*13*
Central & Eastern Europe	488,533	*13*	231	*9*	156,517	*8*	132	*7*	813,002	*18*	685	*16*
Other Western countries	833,827	*22*	610	*25*	452,903	*23*	342	*18*	721,821	*16*	593	*14*
Non-Western	2,185,779	*57*	1,227	*49*	1,073,629	*54*	876	*46*	852,376	*19*	540	*12*
The Middle East	1,183,146	*31*	679	*27*	462,398	*23*	344	*18*	440,127	*10*	275	*6*
Central & Southern America	184,367	*5*	110	*4*	216,703	*11*	185	*10*	157,080	*3*	105	*2*
Sub-Saharan Africa	397,453	*10*	302	*12*	128,146	*6*	126	*7*	92,542	*2*	72	*2*
Asia	420,813	*11*	136	*5*	266,382	*13*	221	*12*	162,627	*4*	88	*2*
Women												
Ancestral Swedes	24,837,500		10,019									
All regions	4,051,642	*100*	1,566	*100*	1,936,951	*100*	802	*100*	4,341,240	*100*	1,990	*100*
Western	1,782,672	*44*	763	*49*	870,545	*45*	431	*54*	3,539,522	*82*	1,763	*89*
Finland	198,023	*5*	165	*11*	209,737	*11*	181	*23*	1,534,156	*35*	847	*43*
Other Nordic	176,584	*4*	72	*5*	101,418	*5*	60	*7*	557,708	*13*	304	*15*
Central & Eastern Europe	335,743	*8*	92	*6*	142,376	*7*	54	*7*	765,823	*18*	317	*16*
Other Western countries	1,072,322	*26*	434	*28*	417,014	*22*	136	*17*	681,835	*16*	295	*15*
Non-Western	2,268,970	*56*	803	*51*	1,066,406	*55*	371	*46*	801,718	*18*	227	*11*
The Middle East	1,028,744	*25*	295	*19*	404,541	*21*	105	*13*	414,007	*10*	113	*6*
Central & Southern America	208,807	*5*	76	*5*	191,892	*10*	63	*8*	146,992	*3*	35	*2*
Sub-Saharan Africa	394,808	*10*	205	*13*	116,323	*6*	56	*7*	89,919	*2*	28	*1*
Asia	636,610	*16*	227	*14*	353,649	*18*	147	*18*	150,800	*3*	51	*3*

Source: author’s calculation based upon the Swedish register data collection “Ageing Well”

Italics were used to differentiate % from absolute numbers in the table

Figure 1 presents hazard ratios by sex for all-cause mortality. The associated regression tables can be found in supplementary Tables S1-S3. At the generation level, **G1** men have an HR of 0.76 (0.73–0.79), indicating a 24% lower risk of mortality than ancestral Swedish men; **G1** women have a 16% lower risk of mortality than ancestral Swedish women (HR = 0.84 [0.80–0.89]). Mortality is elevated among **G1.5** and **G2** men and women; the relative excess is larger among men, especially the **G1.5** (HR = 1.43 [1.36–1.49]). When generation is divided by Western and non-Western-origins, the patterns remain broadly similar to those described at the generation level.

By specific origins, greater variation is observed. Excess mortality among **G1** Finns stand out when all other **G1** display an advantage (HR men = 2.22 [1.97–2.50]; HR women = 1.46 [1.25–1.71]), as do **G1** Sub-Saharan African women (HR = 1.29 [1.12–1.48]). Among the descendants of immigrants, *no origins retain a mortality advantage*. Like the **G1**, excess mortality is found among **G1.5** and **G2** Finland and Sub-Saharan Africa; men and women in these groups display the largest excesses of all descendants. Mortality is consistently elevated in other origin groups except **G1.5** and **G2** other Western men and women. The reversal in mortality advantage in the remaining groups is stark. For example, mortality among **G1** Middle Eastern men is 34% *lower* than ancestral Swedish men, while the mortality of the **G1.5** and **G2** is 24% and 19% *higher* respectively. A comparable intergenerational reversal is evident in all other male groups, apart from **G2** Asia. The same is *broadly* true among women, but there are more cases in which the mortality of descendants women is closer to that of the ancestral Swedes (**G1.5** and **G2** Middle East and Central & Southern America).

**RQ2** “How does mortality from specific causes-of-death vary among the G1, G1.5, and G2 relative to ancestral Swedes, including by origins and sex?”

Table 2 displays the frequency and percentage of deaths from the eight defined cause-of-death categories at the generation and Western non-Western level. The same descriptives by origin can be found in Tables S4 (men) and S5 (women). From Models 1a-c—in which cause-of-death is adjusted for in the analysis of overall mortality for **RQ1**—the leading cause-of-death among men aged 15–44 is suicide (HR = 1.54 [1.48–1.60], relative to cancer), followed by accidents & injuries (HR = 1.27 [1.21–1.32]) and substance use (HR = 1.02 [0.98–1.07]). Cancer (reference) is the leading cause-of-death among women followed by suicide (HR = 0.50 [0.48–0.52]) and other diseases & medical conditions (HR = 0.50 [0.47–0.52]). This highlights the unique risks that men and women face—particularly at young adult ages—and the importance of investigating them separately.

**Table 2:** Deaths by cause, sex, generation, and individual or parental birth region in Sweden, 1997–2016, people aged 15–44

Sex by individual or parental birth region	Cancer		Circulatory diseases		Other diseases		Accidents and injuries		Suicides		Substance use		Other External		Ill-defined		Total deaths
	n	%	n	%	n	%	n	%	n	%	n	%	n	%	n	%	n
Men																	
Ancestral Swedes	2,897	14	2,288	11	2,980	15	3,690	18	4,362	22	2,743	14	701	3	609	3	20,270
Generation 1	453	18	361	15	277	11	333	13	388	16	172	7	167	7	329	13	2,480
Western	208	17	187	15	143	11	173	14	212	17	82	7	68	5	180	14	1,253
Non-Western	245	20	174	14	134	11	160	13	176	14	90	7	99	8	149	12	1,227
Generation 1.5	204	11	161	8	223	12	300	16	389	20	318	17	201	11	109	6	1,905
Western	121	12	110	11	138	13	162	16	191	19	170	17	72	7	65	6	1,029
Non-Western	83	9	51	6	85	10	138	16	198	23	148	17	129	15	44	5	876
Generation 2	426	10	397	9	574	13	713	16	993	23	836	19	248	6	200	5	4,387
Western	389	10	369	10	515	13	625	16	877	23	735	19	180	5	157	4	3,847
Non-Western	37	7	28	5	59	11	88	16	116	21	101	19	68	13	43	8	540
**Total**	**3,980**	**14**	**3,207**	**11**	**4,054**	**14**	**5,036**	**17**	**6,132**	**21**	**4,069**	**14**	**1,317**	**5**	**1,247**	**4**	**29,042**
Women																	
Ancestral Swedes	3,535	35	929	9	1,786	18	976	10	1,671	17	632	6	252	3	238	2	10,019
Generation 1	652	42	138	9	215	14	91	6	184	12	47	3	92	6	147	9	1,566
Western	313	41	68	9	88	12	47	6	108	14	32	4	35	5	72	9	763
Non-Western	339	42	70	9	127	16	44	5	76	9	15	2	57	7	75	9	803
Generation 1.5	226	28	57	7	130	16	71	9	193	24	51	6	34	4	40	5	802
Western	137	32	39	9	71	16	36	8	89	21	26	6	14	3	19	4	431
Non-Western	89	24	18	5	59	16	35	9	104	28	25	7	20	5	21	6	371
Generation 2	549	28	147	7	335	17	197	10	429	22	183	9	62	3	88	4	1,990
Western	504	29	136	8	291	17	168	10	376	21	160	9	55	3	73	4	1,763
Non-Western	45	20	11	5	44	19	29	13	53	23	23	10	7	3	15	7	227
**Total**	**4,962**	**35**	**1,271**	**9**	**2,466**	**17**	**1,335**	**9**	**2,477**	**17**	**913**	**6**	**440**	**3**	**513**	**4**	**14,377**

Frequencies for lowest level origins available in Table S4 (men) and Table S5 (women)

Source: author’s calculation based upon the Swedish register data collection “Ageing Well”

Bold used to highlight total number and % of deaths by sex

Table 3 (men) and Table 4 (women) present results from Models 2a-c (i.e. the cause-specific survival models). The original regression tables can be found in supplementary Tables S6-S10. In Tables 3 and 4, in order to facilitate interpretation of the results, the hazard ratios have been re-estimated so that each cause-of-death among the **G1**, **G1.5** and **G2** becomes relative to the same cause-of-death among ancestral Swedes (as opposed to cancer mortality among ancestral Swedes).

**Table 3:**
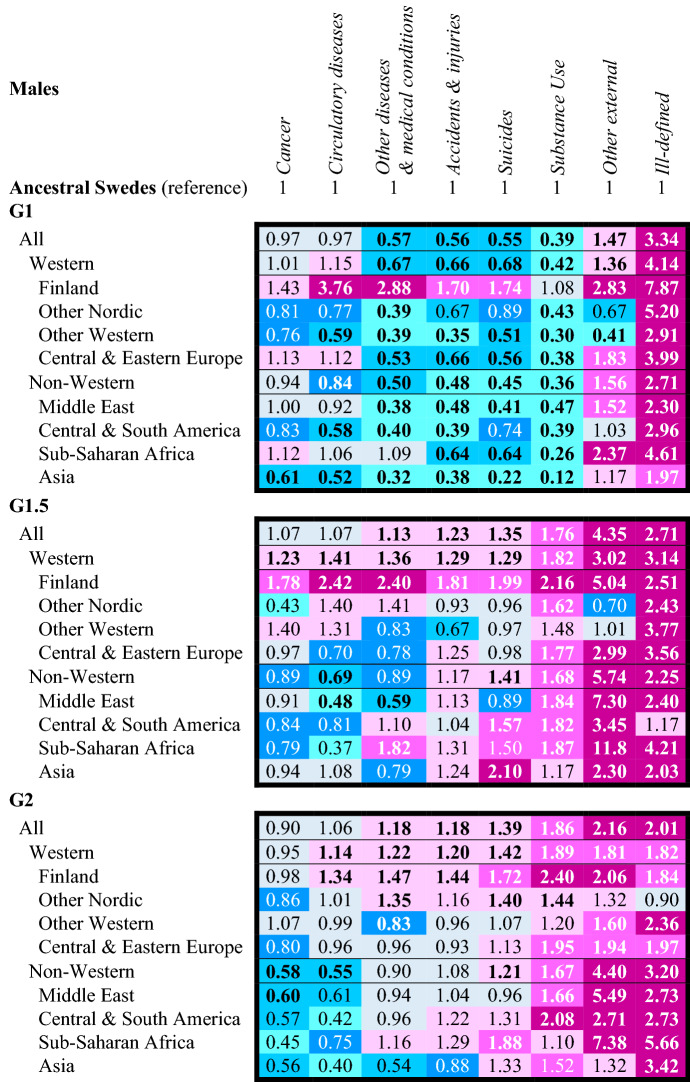
Age and birth cohort adjusted hazard ratios of cause-specific mortality *across* generation, western non-western origins, and lowest level origins in Sweden, 1997–2016, men aged 15–44.

Values significant to p < 0.05 in bold font; colour bands: sky blue HR <  = 0.50; turquoise HR 0.51–0.69; blue HR 0.70–0.89; grey HR 0.90–1.09; light pink HR 1.10–1.49; pink HR 1.50–1.99; dark pink HR >  = 2.00; original extended regression models available in Tables S6-S10

Source: author’s calculation based upon the Swedish register data collection “Ageing Well”

**Table 4:**
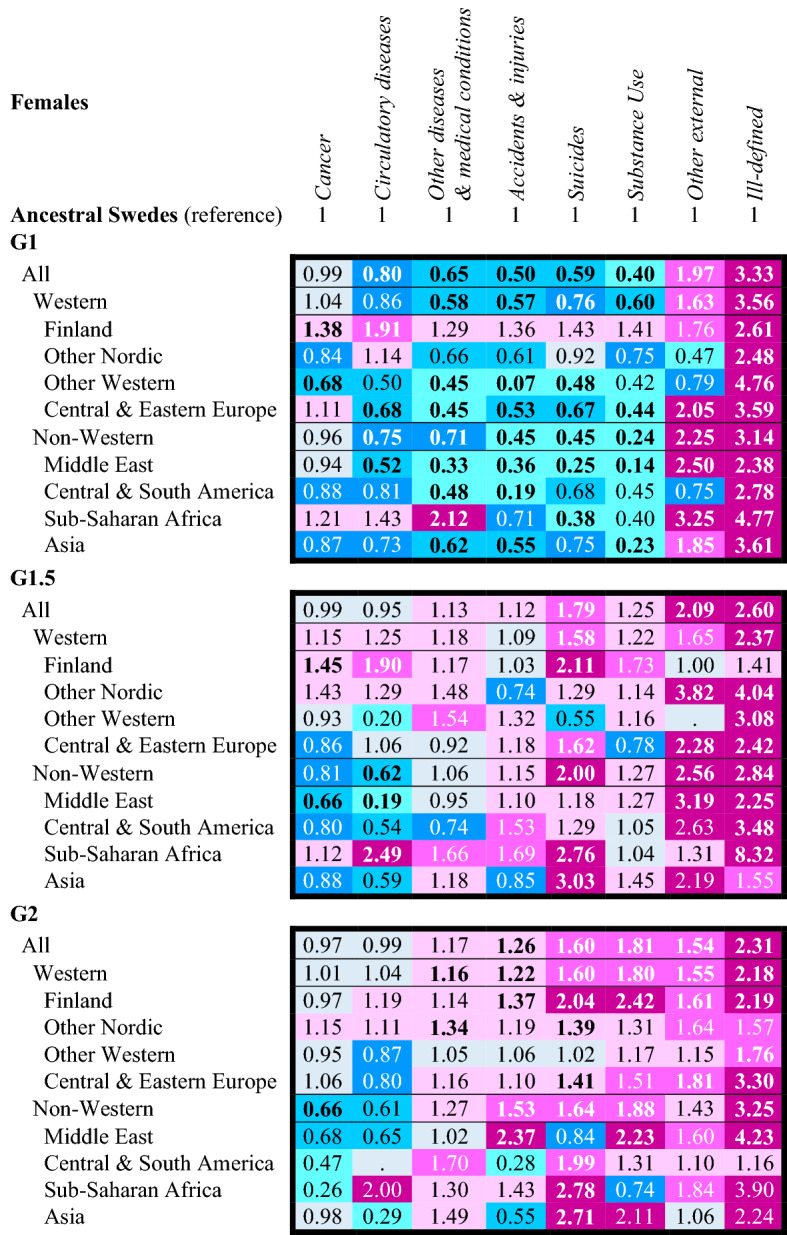
Age and birth cohort adjusted hazard ratios of cause-specific mortality across generation, western non-western origins, and lowest level origins in Sweden, 1997–2016, women aged 15–44

Values significant to p < 0.05 in bold font; colour bands: sky blue HR <  = 0.50; turquoise HR 0.51–0.69; blue HR 0.70–0.89; grey HR 0.90–1.09; light pink HR 1.10–1.49; pink HR 1.50–1.99; dark pink HR >  = 2.00; original extended regression models available in Tables S6-S10

Source: author’s calculation based upon the Swedish register data collection “Ageing Well”

Across sexes and origins, **G1** men and women tend to have lower mortality from most cause-of-death relative to the ancestral Swedes. Mortality is elevated from *other external causes-of-death* in all groups except other Nordic, other Western and Central & Southern America men and women. **G1** mortality is also systematically elevated for *ill-defined causes-of-death* (often HR > 2.00). Mortality among the **G1** is especially low for *other diseases and medical conditions*, *accidents & injuries*, *suicide* and *substance use*. **G1** Finns and **G1** Sub-Saharan Africans are the main exceptions. **G1** Finnish men and women have elevated mortality from each cause-of-death, particularly *circulatory diseases* (men HR = 3.76 [2.85–4.95]; women HR = 1.91 [1.23–2.98]). The cause-of-death pattern for **G1** Sub-Saharan African men and women is distinctive. Comparable to the other **G1**, they have lower mortality from *accidents and injuries*, *suicide* and *substance use*. However, this coincides with high mortality from *cancer*, *circulatory diseases* and *other diseases and medical conditions*. **G1** women from Sub-Saharan African experience a particularly high risk of mortality from *other diseases & medical conditions* (HR = 2.12 [1.64–2.74]).

The cause-of-death patterns among the **G1.5** and **G2** differ markedly from the **G1**. They display excess mortality in all four external cause categories. Among men, the excess in other *external cause mortality* is very high, particularly among non-Western **G1.5** (HR = 5.74 [4.75–6.92]) and non-Western **G2** (HR = 4.40 [3.43–5.64])—**G1.5** and **G2** Sub-Saharan African Middle Eastern men have HRs *exceeding five*. By origins, substance use mortality is also typically 50% higher among **G1.5** and **G2** men across origins. The excesses in *accident & injury* and *suicide* are smaller—and in some cases non-existent (e.g. **G1.5** and **G2** Middle Eastern men). Yet, some origins have specific risks. See e.g. the elevated suicide mortality of **G1.5** Finnish, **G1.5** Asian and **G2** Sub-Saharan African men and high accident & injury mortality of **G1.5** and **G2** Finnish men.

Among **G1.5** and **G2** women, conversely, their relative excess in *suicide* mortality stands out (**G1.5** HR = 1.79 [1.54–2.07]; **G2** HR = 1.60 [1.44–1.78]); **G1.5** and **G2** Finnish, Sub-Saharan African and Asian women and **G2** Central & Southern American women have HRs that *at least twice as high* as ancestral Swedish women. Unlike among men, there are differences between the **G1.5** and **G2** among women in their external mortality. Among the **G1.5**, mortality from *other external causes* is more elevated (Western HR = 1.65 [0.96–2.82]; non-Western HR = 2.56 [1.62–4.03]), while among **G2** women it is instead *substance use* mortality (Western HR = 1.80 [1.51–2.14]; non-Western HR = 1.88 [1.24–2.85]) that is more elevated alongside mortality from suicide. Like **G1.5** and **G2** men, the relative excess mortality in accident & injuries tends to be smaller.

The patterns for *cancers*, *circulatory diseases* and *other diseases and medical conditions* are less regularised among **G1.5** and **G2** men and women. At the Western non-Western level, Western-origin **G1.5** and **G2** tend to have higher mortality from these causes-of-death, while **G1.5**. Non-Western **G1.5** and **G2** tend to have lower mortality risks—except for *other diseases and medical conditions*. However, at the lowest level, there is a substantial variation across sexes and origins. For example, in the elevated circulatory disease mortality of both **G1.5** and **G2** Sub-Saharan African women (**G1.5** HR = 2.49 [1.18–5.23]; **G2** HR = 2.00 [0.75–5.34]). Comparable to the **G1**, mortality is elevated among both **G1.5** and **G2** men and women from *ill-defined causes-of-death*.

**RQ3** “How does the variation in cause-specific mortality combine to produce the all-cause mortality differentials observed among the G1, G1.5, and G2 relative to ancestral Swedes?”

**Fig. 2:**
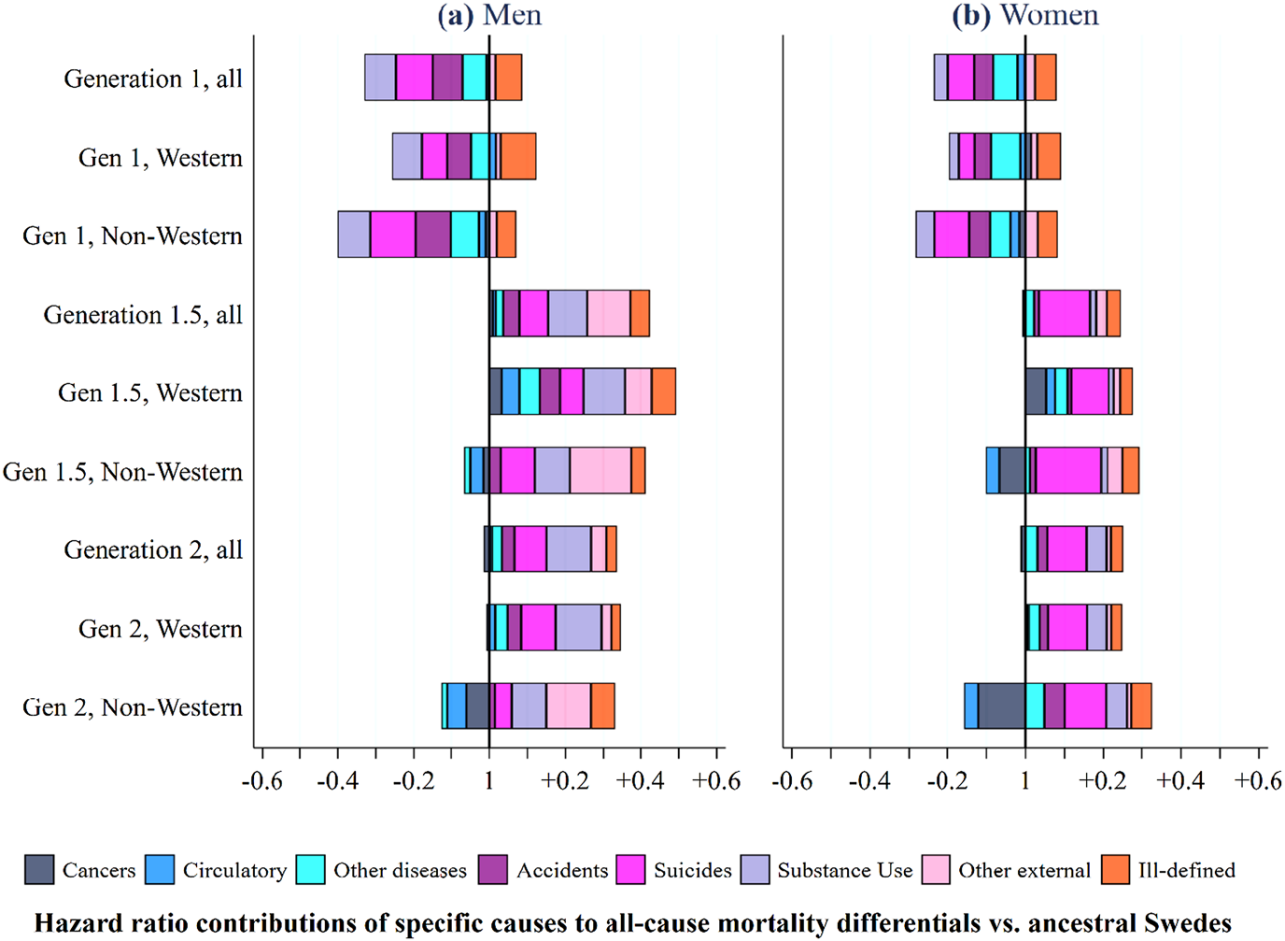
Hazard ratio contributions of causes-of-death to total mortality differences relative to ancestral Swedes, Sweden, 1997–2016, people aged 15–44. Source: author’s calculation based upon the Swedish register data collection “Ageing Well”

Figure 2 shows how the variation in mortality from specific causes-of-death (Tables 3 and 4) among the **G1**, **G1.5** and **G2** combines to generate the all-cause mortality differences relative to ancestral Swedes (Fig. 1). This is made possible through the extended survival approach. How to derive these “contributions”—using the original regression models from Tables S6-S10 is outlined in the supplementary materials. Figure 2 shows results at the generation and western and non-western level. The same panel relating to more granular origins is available in Figure S3.

From Fig. 2, the low all-cause mortality of **G1** men and women at the generational level—relative to ancestral Swedes—is driven by three of the four external categories (*accidents & injuries*, *suicide*, and *substance use*) and *other diseases and medical conditions*. Patterns are similar at the Western, non-Western level. For **G1.5** and **G2** men and women, this pattern is reversed. Their high all-cause mortality—relative to ancestral Swedes—is driven by excess mortality in *all* of the external categories, with some contribution of *other diseases & medical conditions*. *Suicide* plays a major role in the excess mortality of **G1.5** and **G2** women, especially **G1.5** non-Western. *Substance use* and *other external causes* are the driving forces among **G1.5** and **G2** men. In Figure S3, these broader generational patterns are largely reflected in specific origins except for **G1** Finns (every cause contributes to an excess) and **G1** Sub-Saharan African women (*other diseases and medical conditions* are a key contributor to their overall excess mortality). For some **G1.5** and **G2**, excess mortality in one specific cause almost fully accounts for an overall mortality excess (e.g. suicide in **G1.5** Asian men and women).

## Discussion

This study set out to investigate all-cause and cause-specific mortality among immigrants and the descendants of immigrants in Sweden with respect to the “migrant mortality advantage” and an emerging body of evidence charting its reversal among the **G1.5** and **G2**. The study’s two main aims were to (1) describe intergenerational, all-cause mortality differentials between immigrants and the descendants of immigrants relative to ancestral Swedes and (2) identify the causes-of-death driving any differences. To achieve the two aims, three research questions were outlined.

**RQ1** asked “*How does all-cause mortality vary among the G1, G1.5, and G2 relative to ancestral Swedes, including by origins and sex?*” A migrant mortality advantage was found in all **G1** groups except for men and women from Finland and women from Sub-Saharan Africa, who had excess mortality. Excess mortality was found among nearly all **G1.5** and **G2** groups except for those with other Western origins—relative excesses were particularly strong among men. **RQ2** asked “*How does mortality from specific causes-of-death vary among the G1, G1.5, and G2 relative to ancestral Swedes, including by origins and sex?*” The **G1** had lower mortality than ancestral Swedes across causes-of-death, particularly *other diseases & medical conditions*, *accidents & injuries*, *suicide* and *substance use*. The opposite was largely true for the **G1.5** and **G2**; their mortality was highly elevated from *suicide* (among women) and *substance use* and *other external causes* (among men). There was a broad consistency in these patterns by sex and generation, with variation by specific origins. **RQ3** asked “*How does the variation in cause-specific mortality combine to produce the all-cause mortality differentials observed among the G1, G1.5, and G2 relative to ancestral Swedes?*” External cause-of-death mortality was driving both the migrant migrant mortality advantage of the **G1** and the descendant mortality disadvantage of the **G1.5** and **G2**.

The findings fell in line with expectations based upon the literature. They can be placed in the context of research in Europe that documents the reversal of the migrant mortality advantage between generations of immigrants and their descendants (De Grande et al., 2014; Guillot et al., 2019; Khlat et al., 2019; Manhica et al., 2015; Mehta et al., 2019; Singh & Siahpush, 2001; Tarnutzer et al., 2012; Vandenheede et al., 2014, 2015; Wallace, 2016). To this research, the article contributes new empirical evidence documenting the causes-of-death driving this intergenerational reversal in “migrant mortality advantage” in young adulthood, alongside highlighting the precarious mortality situation of immigrants who arrive as children (the **G1.5**).

Patterns for the **G1** are largely consistent with the “healthy migrant effect”. For example, lower mortality among other Western immigrants could be interpreted in terms of reason for arrival (tertiary education and highly skilled occupations) (Eurostat, 2021). Lower mortality among non-Western immigrants could be interpreted in terms of the increased physical and cultural distance to Sweden and presence of additional migration barriers (Chiswick et al., 2008; Shor & Roelfs, 2021). Higher mortality among Finnish immigrants, on the other hand, is consistent with *negative* selection effects due to the ease of access of Finnish migrants to Sweden, the close cultural proximity of the two countries, and the main reason for arrival (at least traditionally, to fill national demand in unskilled labour). Across all **G1** origins, excepts Finns, low mortality from *accidents & injuries*, *suicide* and *substance use* is consistent with the concept of a “migrant” personality (Boneva & Frieze, 2001), alongside empirical evidence that migrants have lower risk-seeking attitudes than ancestral natives do (Bonin et al., 2009). For the **G1.5** and **G2**, it seems unlikely that a lack of selection effects *alone* would generate their elevated mortality, especially for origins in which the **G1** have such large mortality advantages (Asia, Central & Southern America, Middle East). Additionally, it is hard to reconcile an explanation that focuses on health with mortality disadvantages driven by external causes-of-death. Moreover, even if the **G1.5** and **G2** do not subscribe to the concept of a “migrant” personality, the (limited) empirical evidence suggests that their risk-seeking attitudes resemble ancestral natives (Bonin et al., 2009).

Similarly, it seems unlikely that acculturation among the descendants of immigrants—away from the prevailing health behaviours of their parents’ origin country and toward those of the host country—provide the driving force behind a reversal in mortality at young adult ages where the main causes-of-death are external and unrelated to physical health. In fact, among the **G1.5** and **G2**—notably non-Westerns—cancer and circulatory diseases are considerably lower than among the ancestral Swedes. This *might* indicate some positive intergenerational transmission of health behaviours. It *might* also indicate that the **G1.5** and **G2** are dying from external causes-of-death before these diseases can take hold. Nevertheless, there could be evidence of intergenerational transmission of cultural norms for specific origins. For example, regarding the low suicide mortality in the **G1.5** and **G2** Middle East. This group comprises Islamic countries for which strict religious sanctions against suicide exist (Shah & Chandia, 2010). Unlike other descendant groups, which have elevated suicide mortality, the mortality of the **G1.5** and **G2** Middle East is similar to—or lower than—ancestral Swedes.

The findings *are* consistent with how psychosocial factors might be expected to affect variation in causes-of-death between the **G1**, **G1.5** and **G2**. A reversal (i.e. from mortality advantage to disadvantage across generations) was most consistently documented—across origins and sexes—in mortality from *accidents & injuries*, *suicide*, *substance use* and *other external causes-of-death*, causes typically associated with psychosocial factors. The **G1.5** and **G2** represent an age-specific vulnerability to the challenges of migration (e.g. racism and discrimination). This is compounded by the change in reference group between generations (leading to more negative evaluations of their life situation) and the (often) high expectations of migrant parents. Such factors may well result in increased stress, hostility, depression, hopelessness and risk-seeking behaviours that produce a reversal in external mortality that not only erodes, but *reverses* the mortality advantage of the **G1**. Sub-Saharan Africans—for which all three generations of have sizeable mortality risks—may encounter especially profound challenges. A recent report revealed that anti-black racism and discrimination strongly structure conditions in the Swedish labour market for Sub-Saharan Africans, who are very disadvantaged with respect to education-occupational mismatch, career opportunities and unemployment (Wolgast et al., 2018). Sub-Saharan Africans are also seen as being most vulnerable to hate crimes (including physical violence) and “everyday racism” in Sweden (Mångkulturellt Centrum, 2014). Such instances, according to a qualitative study, have led to a loss of trust in society, a sense of uselessness (due to an inability to provide for family) and social isolation (Osueke, 2020).

While the analysis did not adjust childhood or adult SEP, the findings might well reflect the greater socioeconomic disadvantage of the **G1.5** and **G2** compared to ancestral Swedes. That external cause mortality drives their high excess mortality is consistent with the idea that external causes make a large contribution to young adult socioeconomic differences in mortality (Rosvall et al., 2006). They are also consistent with the idea that poor childhood conditions are linked to high external cause mortality (Galobardes et al., 2004, 2006, 2008; Montez & Hayward, 2011). Prior research on a combined **G1.5** and **G2** in Sweden showed that excess all-cause and external mortality among Finnish, Balkan, Middle Eastern and other non-European men attenuated after adjusting income and education, though some excesses persisted (Manhica et al., 2015). This indicates that either adult SEP cannot account for all of their excess mortality or that income or education do not capture all aspects of their adult social disadvantage. The patterns in the detailed external-cause-of-death categories here could offer some insight. For example, among Finnish men, who are concentrated in manufacturing, construction and recycling (Englund, 2003), adverse working conditions might contribute to the excess *accident & injury* mortality of all three generations of Finns. Poor housing conditions might also play a role in accidents and injuries in the home. The systematic excess mortality in *other external causes*—largely homicides and deaths of undetermined intent—might relate to residential segregation, a process linked with an increased risk of violence (Light & Thomas, 2019). Very high other external mortality is found in the non-Western groups—including all three generations of Sub-Saharan African and Middle Eastern men and women—these are origins known to be especially segregated in Sweden (Malmberg et al., 2018). Among the **G1**, specifically non-Westerns, discussion of their mortality advantage with respect to SEP is complicated by their lower SEP compared to ancestral Swedes—and indeed the **G2**—a well-known paradox attributed to protective in-selection effects and cultural factors (Guillot et al., 2019).

Previous research rules out a *salmon bias effect* in the Nordic countries (Andersson & Drefahl, 2017; Dunlavy et al., 2022; Norredam et al., 2015). Furthermore, previous research has only documented a limited effect of missing emigration dates (the main *data artefact* relevant to this study) on the size of the “migrant mortality advantage” of the **G1** in Sweden (Wallace & Wilson, 2020). It is also relevant that the deaths of residents abroad are captured in the Swedish cause-of-death register, even if they cannot always be assigned an ICD-code (Brooke et al., 2017). This likely accounts for the higher mortality ratios from *ill-defined* causes in the **G1** and **G1.5**).

Although the all-cause mortality results are consistent with wider European research, the ability to generalise them might be affected by contextual factors that are specific to Sweden. First, the presence of an extensive, socio-democratic welfare state that practices an integration policy of *inclusive multiculturalism*—as compared to countries with other types of welfare model and/or integration policies (such as *assimilation*) —and how this might directly and indirectly affect mortality through other domains of life (e.g. health, education, housing, and the labour market). Second, how the national mortality situation of Sweden might differ from elsewhere, including whether specific causes-of-death are more or less prominent in the age range studied. Third, a migration history driven by intra-Nordic and humanitarian migration—as compared to countries with migration flows characterised by migration from e.g. former colonies. It might be that there are factors specific to the unique experiences of arriving as—or being a descendant of—a refugee immigrant or immigrant from a former colony that affect their total and cause-specific mortality risks.

There are limitations to this study. First, the analysis covers a unique age range of mortality in which mortality from external causes drives overall mortality differences between groups. As such, the conclusions from this study cannot be applied to observed intergenerational mortality differentials at older adult ages (in which behaviour-related chronic diseases dominate) or across the entire adult age range either in Sweden or elsewhere. Second, due to the age range studied, it was not possible to analyse natural causes-of-death in more detail. The hazard ratios for cancer, circulatory diseases and other diseases & medical conditions likely mask substantial variation in mortality from granular causes. It is know, for example, that certain migrant groups have elevated mortality from specific cancer sites. Third, the study has not accounted for important socioeconomic health inequalities that might attenuate—or even account for—the observed mortality differences. The type of estimates provided are critical to understanding the world “as it is”. However, the ability to draw direct policy conclusions might have been better facilitated by adjustment for relevant socioeconomic indicators.

Ultimately, the systematicity of the excess mortality observed among the **G1.5** and **G2** in this study should represent a major social and public health concern in Sweden. Mortality is the most fundamental of all life’s inequalities; every other type of inequality is contingent upon being alive (Raalte et al., 2018). Immigrants who arrive in a new country do so with hopes for a better future for their children. This hope—at least with respect to expectations of life—has so far failed to materialise in Sweden. Of particular concern is that the excess mortality of young adult **G1.5** and **G2** is generated almost exclusively by external causes-of-death considered to be preventable. Mortality in the age range studied is also premature—with decades of potential life lost (OECD, 2019). The findings call into question the effectiveness of Sweden’s national integration policy—a model celebrated around the world as *the* model for positive multicultural immigrant integration (Borevi, 2014). They can also help to inform public health policy, specifically national action programmes for suicide (Wasserman, 2021) and drug use (Ministry of Health & Social Affairs, 2014) prevention. Such programmes cite the enhanced vulnerability of socially disadvantaged groups (of which there is overlap with the **G1.5** and **G2**) and men, but make little to no mention of the descendants of immigrants. This study thus highlights the need for greater intersectionality in Swedish public health policy by providing new, reliable and granular evidence highlighting the **G1.5** and **G2** (and specific origins) as especially vulnerable groups that urgently require targeted intervention policies to reduce their mortality from these, and other, external causes-of-death.

## Supplementary Information

Below is the link to the electronic supplementary material.

Supplementary file1 (PDF 933 KB)

## Data Availability

Swedish register data collection “Ageing Well” is only available to approved researchers.
