# Supplementary material for: Mortality Advantage Reversed: The Causes of Death Driving All-Cause Mortality Differentials Between Immigrants, the Descendants of Immigrants and Ancestral Natives in Sweden, 1997–2016
Source: Eur J Popul. 2022 Oct 27;38(5):1213–41. doi: 10.1007/s10680-022-09637-0 (PMC9727037; doi:10.1007/s10680-022-09637-0)
Supplement: Supplementary file 1 — Supplementary file1 (PDF 933 KB) [file 10680_2022_9637_MOESM1_ESM.pdf]

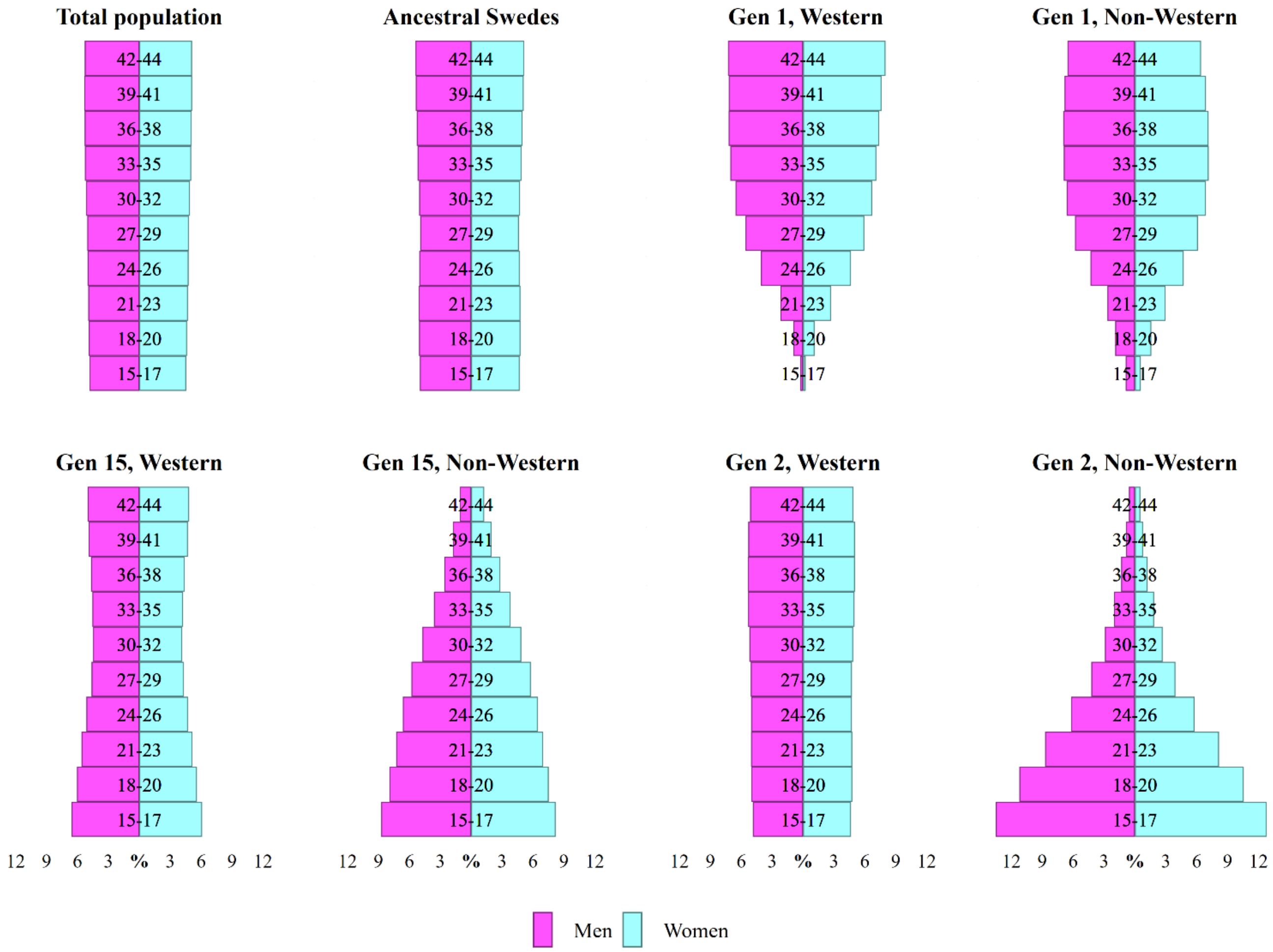

Figure S1. Population age distributions, generation by western and non-western origins, Sweden, 1997-2016.

Source: author’s calculation based upon the Swedish register data collection “Ageing Well”

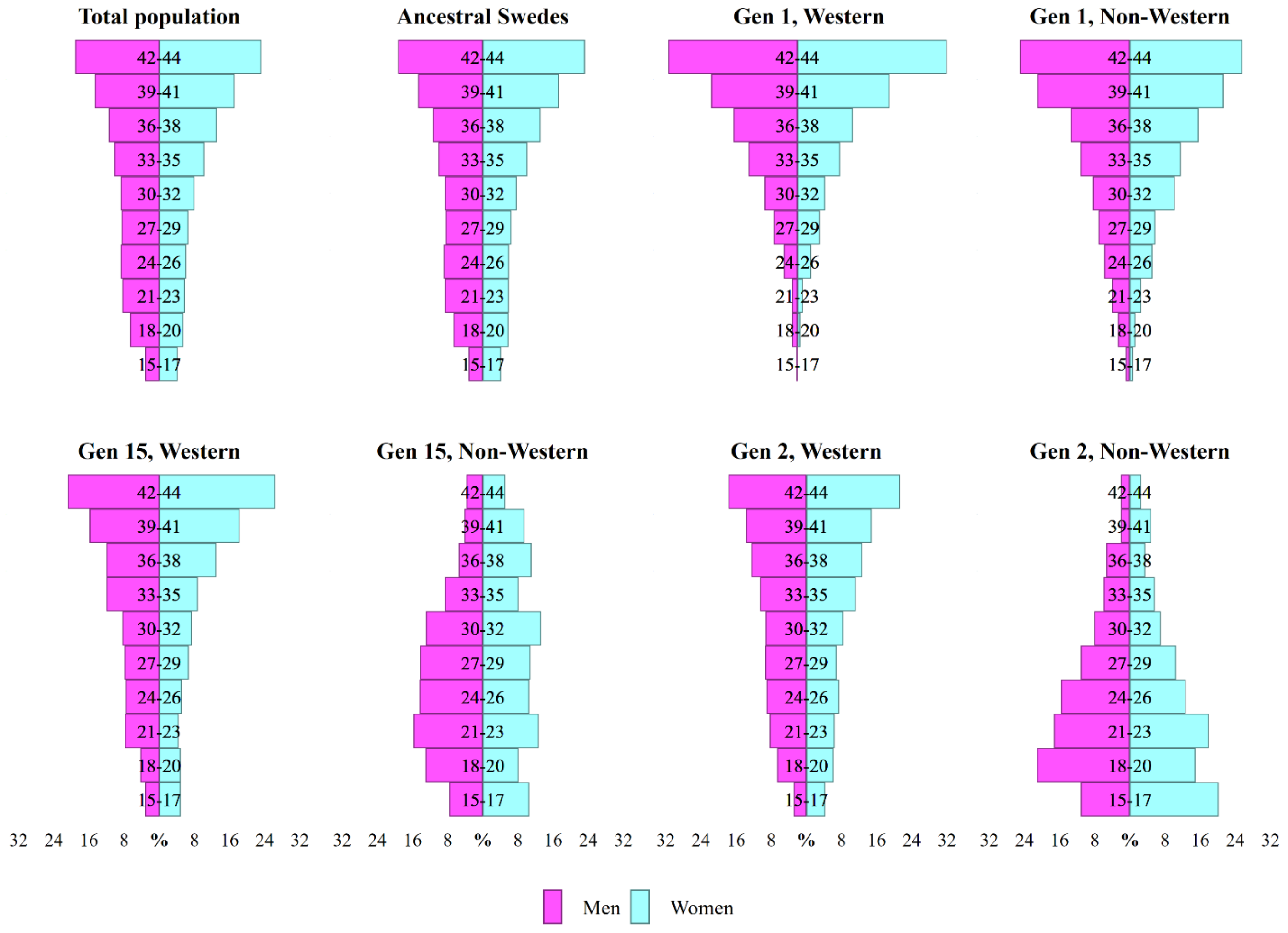

Figure S2. Age-at-death distributions, generation by western and non-western origins, Sweden, 1997-2016.
Source: author's calculation based upon the Swedish register data collection "Ageing Well"

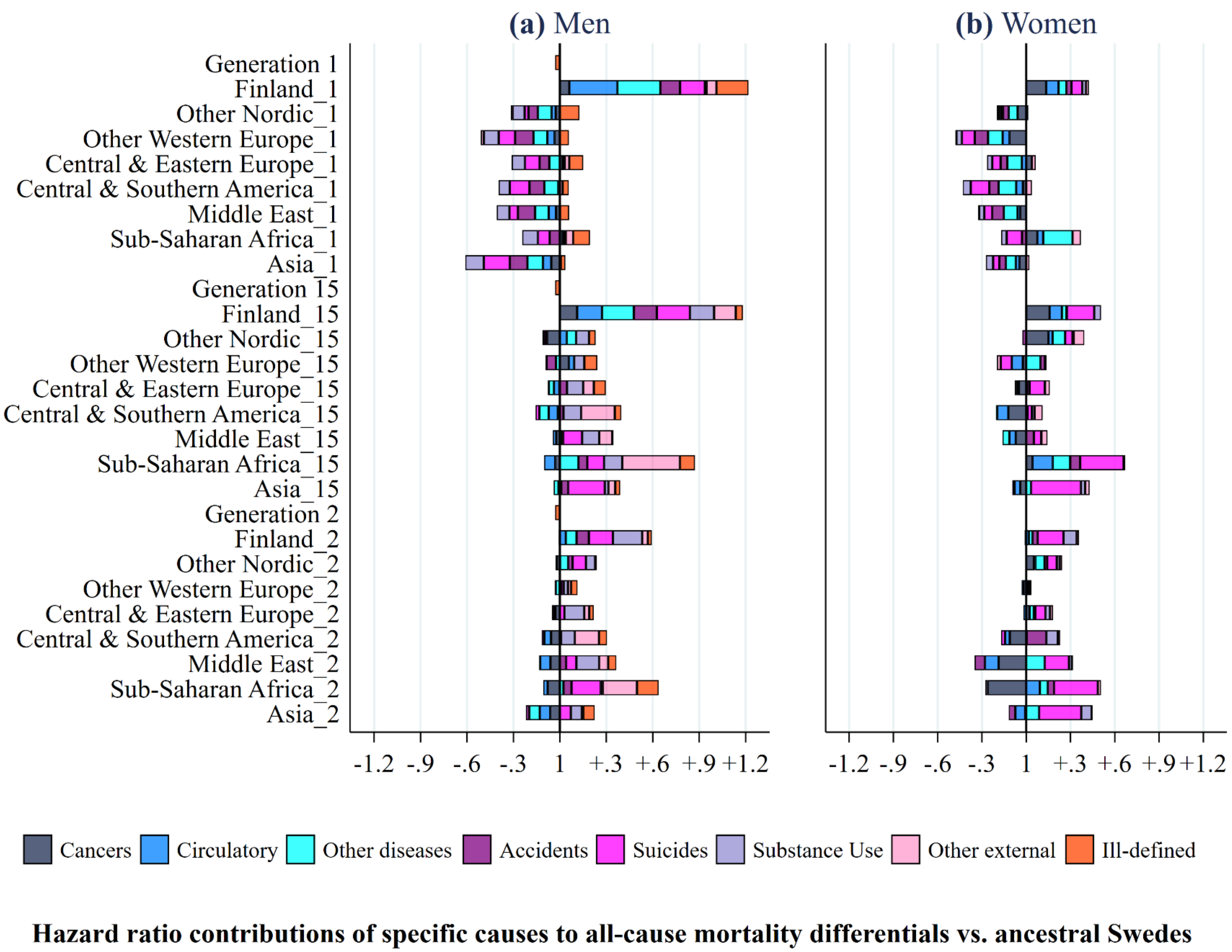

Figure S3. Contributions of specific causes of death to all-cause mortality differences between the **G1**, **G1.5**, **G2** and ancestral Swedes, lowest level origins, ages 15-44, 1997-2016.

Source: author's calculation based upon the Swedish register data collection "Ageing Well"

Table S1. Model 1a, extended, competing-risks survival model, people aged 15-44, 1997-2016, generational level.

**Model 1a**	**Men**					**Women**				
	HR	Sig.	95%CIs			HR	Sig.	95%CIs		
**Generation**										
Ancestral Swedes	**1**									
Generation 1	0.76	**	0.72	-	0.79	0.84	**	0.80	-	0.89
Generation 1.5	1.42	**	1.36	-	1.49	1.24	**	1.15	-	1.33
Generation 2	1.32	**	1.28	-	1.37	1.24	**	1.18	-	1.30
**Birth year**	0.99	**	0.98	-	0.99	0.98	**	0.98	-	0.99
**Cause of death**										
Cancer	**1**									
Circulatory diseases	0.81	**	0.77	-	0.84	0.26	**	0.24	-	0.27
Other diseases & medical conditions	1.02		0.98	-	1.06	0.50	**	0.47	-	0.52
Accidents & injuries	1.27	**	1.21	-	1.32	0.27	**	0.25	-	0.29
Suicide	1.54	**	1.48	-	1.60	0.50	**	0.48	-	0.52
Substance Use	1.02		0.98	-	1.07	0.18	**	0.17		0.20
Other external causes-of-death	0.33	**	0.31	-	0.35	0.09	**	0.08	-	0.10
Ill-defined	0.31	**	0.29	-	0.33	0.10	**	0.09	-	0.11

Notes: p<0.01 **; p<0.05 *; p<0.1 +
Source: author's calculation based upon the Swedish register data collection "Ageing Well"

Table S2. Model 1b, extended, competing-risks survival model, people aged 15-44, 1997-2016, generation by western, non-western origins.

**Model 1b**	**Men**					**Women**				
	HR	Sig.	95%CIs			HR	Sig.	95%CIs		
**Generation**										
Ancestral Swedes	**1**									
Generation 1										
Western	0.87	**	0.82	-	0.92	0.89	**	0.83	-	0.96
Non-Western	0.67	**	0.63	-	0.71	0.80	**	0.74	-	0.86
Generation 1.5										
Western	1.49	**	1.40	-	1.59	1.28	**	1.16	-	1.41
Non-Western	1.35	**	1.26	-	1.44	1.19	**	1.07	-	1.32
Generation 2										
Western	1.34	**	1.30	-	1.39	1.25	**	1.19	-	1.31
Non-Western	1.21	**	1.11	-	1.32	1.17	*	1.02	-	1.34
**Birth year**	0.99	**	0.98	-	0.99	0.98	**	0.98	-	0.99
**Cause of death**										
Cancer										
Circulatory diseases	0.81	**	0.77	-	0.84	0.26	**	0.24	-	0.27
Other diseases & medical conditions	1.02		0.98	-	1.06	0.50	**	0.47	-	0.52
Accidents & injuries	1.27	**	1.21	-	1.32	0.27	**	0.25	-	0.29
Suicide	1.54	**	1.48	-	1.60	0.50	**	0.48	-	0.52
Substance Use	1.02		0.98	-	1.07	0.18	**	0.17	-	0.20
Other external causes-of-death	0.33	**	0.31	-	0.35	0.09	**	0.08	-	0.10
Ill-defined	0.31	**	0.29	-	0.33	0.10	**	0.09	-	0.11

Notes: p<0.01 **; p<0.05 *; p<0.1 +
Source: author's calculation based upon the Swedish register data collection "Ageing Well"

Table S3. Model 1c, extended, competing-risks survival model, people aged 15-44, 1997-2016, generation by lowest level origins.

**Model 1c**	**Men**					**Women**				
	HR	Sig.	95%CIs			HR	Sig.	95%CIs		
**Generation**										
Ancestral Swedes	**1**									
Generation 1										
Finland	2.22	**	1.97	-	2.50	1.46	**	1.25	-	1.71
Other Nordic	0.81	*	0.69	-	0.96	0.85		0.67	-	1.07
Other Western	0.55	**	0.48	-	0.62	0.61	**	0.50	-	0.75
Central & Eastern Europe	0.84	**	0.77	-	0.91	0.86	**	0.78	-	0.95
The Middle East	0.66	**	0.61	-	0.71	0.64	**	0.57	-	0.72
Central & Southern America	0.65	**	0.54	-	0.79	0.72	**	0.57	-	0.90
Sub-Saharan Africa	0.95		0.85	-	1.07	1.29	**	1.12	-	1.48
Asia	0.42	**	0.36	-	0.50	0.81	**	0.71	-	0.92
Generation 1.5										
Finland	2.18	**	1.99	-	2.39	1.52	**	1.31	-	1.76
Other Nordic	1.12		0.91	-	1.37	1.44	**	1.12	-	1.86
Other Western	1.15		0.97	-	1.36	0.99		0.76	-	1.29
Central & Eastern Europe	1.22	**	1.10	-	1.36	1.12		0.94	-	1.32
The Middle East	1.24	**	1.11	-	1.38	0.94		0.77	-	1.13
Central & Southern America	1.30	**	1.12	-	1.50	1.04		0.81	-	1.34
Sub-Saharan Africa	1.77	**	1.49	-	2.11	1.84	**	1.42	-	2.40
Asia	1.35	**	1.18	-	1.54	1.35	**	1.15	-	1.59
Generation 2										
Finland	1.59	**	1.52	-	1.67	1.38	**	1.28	-	1.48
Other Nordic	1.22	**	1.12	-	1.32	1.26	**	1.12	-	1.41
Other Western	1.08	*	1.00	-	1.17	1.02		0.91	-	1.14
Central & Eastern Europe	1.17	**	1.08	-	1.27	1.22	**	1.08	-	1.37
The Middle East	1.19	**	1.05	-	1.34	1.14		0.94	-	1.37
Central & Southern America	1.23	*	1.02	-	1.49	0.97		0.69	-	1.35
Sub-Saharan Africa	1.53	**	1.21	-	1.93	1.30		0.90	-	1.88
Asia	1.01		0.82	-	1.24	1.36	*	1.03	-	1.79
**Birth year**	0.99	**	0.99	-	0.99	0.98	**	0.98	-	0.99
**Cause of death**										
Cancer										
Circulatory diseases	0.81	**	0.77	-	0.84	0.26	**	0.24	-	0.27
Other diseases & medical conditions	1.02		0.98	-	1.06	0.50	**	0.47	-	0.52
Accidents & injuries	1.27	**	1.21	-	1.32	0.27	**	0.25	-	0.29
Suicide	1.54	**	1.48	-	1.60	0.50	**	0.48	-	0.52
Substance Use	1.02		0.98	-	1.07	0.18	**	0.17	-	0.20
Other external causes-of-death	0.33	**	0.31	-	0.35	0.09	**	0.08	-	0.10
Ill-defined	0.31	**	0.29	-	0.33	0.10	**	0.09	-	0.11

Notes: p<0.01 **; p<0.05 *; p<0.1 +

Source: author's calculation based upon the Swedish register data collection "Ageing Well"

Table S4. Deaths by cause among generations by lowest level origins, men.

**Men**	*Cancer*	*Circulatory diseases*	*Other diseases & medical conditions*	*Accidents & Injuries*	*Suicides*	*Substance Use*	*Other External*	*Ill-defined*
**Generation 1**								
Finland	25	52	52	38	46	18	12	29
Other Nordic	20	15	10	21	33	10	<5	27
Other Western	46	28	24	27	46	17	6	37
Central & Eastern Europe	117	92	57	87	87	37	46	87
The Middle East	146	106	58	89	90	65	54	71
Central Southern America	20	11	10	12	27	9	6	15
Sub-Saharan Africa	51	38	51	37	44	11	26	44
Asia	28	19	15	22	15	5	13	19
**Generation 1.5**								
Finland	54	58	75	70	91	62	37	16
Other Nordic	<5	13	17	14	17	18	<5	6
Other Western	23	17	14	14	24	23	<5	13
Central & Eastern Europe	39	22	32	64	59	67	29	30
The Middle East	36	15	24	57	53	69	70	20
Central Southern America	17	13	23	27	48	35	17	5
Sub-Saharan Africa	8	<5	19	17	23	18	29	9
Asia	22	20	19	37	74	26	13	10
**Generation 2**								
Finland	175	188	270	326	462	404	89	69
Other Nordic	59	55	96	102	145	94	22	13
Other Western	97	71	77	111	146	103	35	45
Central & Eastern Europe	58	55	72	86	124	134	34	30
The Middle East	20	16	32	44	48	52	44	19
Central Southern America	7	<5	12	19	24	24	8	7
Sub-Saharan Africa	<5	<5	8	11	19	7	12	8
Asia	7	<5	7	14	25	18	<5	9

Notes: cells with less than 5 events anonymised to comply with terms and conditions of data supplier; CAN = cancer; CIR = circulatory diseases; DIS = other diseases & medical conditions; ACC = accidents; SUI = suicides; EXT = other external causes of death; ILL = ill-defined causes; TOT = total (as rows or columns)
Source: author's calculation based upon the Swedish register data collection "Ageing Well"

Table S5. Deaths by cause among generations by lowest level origins, women.

**Women**	*Cancer*	*Circulatory diseases*	*Other diseases & medical conditions*	*Accidents & Injuries*	*Suicides*	*Substance Use*	*Other External*	*Ill-defined*
**Generation 1**								
Finland	55	20	26	15	27	10	5	7
Other Nordic	25	9	10	5	13	<5	<5	5
Other Western	36	7	12	<5	12	<5	<5	17
Central & Eastern Europe	197	32	40	26	56	14	26	43
The Middle East	152	22	27	16	19	<5	29	26
Central Southern America	33	8	9	<5	12	<5	<5	7
Sub-Saharan Africa	68	21	60	11	10	<5	13	18
Asia	86	19	31	15	35	<5	13	24
**Generation 1.5**								
Finland	61	21	25	12	42	13	<5	<5
Other Nordic	21	5	11	<5	9	<5	<5	<5
Other Western	18	<5	15	7	5	<5	<5	<5
Central & Eastern Europe	37	12	20	14	33	6	7	7
The Middle East	26	<5	19	12	22	9	9	6
Central Southern America	17	<5	8	9	13	<5	<5	5
Sub-Saharan Africa	12	7	9	5	14	<5	<5	6
Asia	34	6	23	9	55	10	6	<5
**Generation 2**								
Finland	211	68	125	82	210	94	25	32
Other Nordic	98	25	58	28	56	20	10	9
Other Western	104	25	58	32	53	23	9	13
Central & Eastern Europe	91	18	50	26	57	23	11	19
The Middle East	24	6	18	23	14	14	<5	10
Central Southern America	6	<5	11	<5	12	<5	<5	<5
Sub-Saharan Africa	<5	<5	5	<5	10	<5	<5	<5
Asia	13	<5	10	<5	17	5	<5	<5

Notes: cells with less than 5 events anonymised to comply with terms and conditions of data supplier; CAN = cancer; CIR = circulatory diseases; DIS = other diseases & medical conditions; ACC = accidents; SUI = suicides; EXT = other external causes of death; ILL = ill-defined causes; TOT = total (as rows or columns)
Source: author's calculation based upon the Swedish register data collection "Ageing Well"

Table S6. Model 2a, extended, competing-risks survival model, people aged 15-44, 1997-2016, generational level, cancer among ancestral Swedes as reference.

**Model 2a**	**Men**					**Women**				
	HR	Sig.	95%CIs			HR	Sig.	95%CIs		
**Generation by cause**										
Cancer										
Ancestral Swedes	**1**					**1**				
Generation 1	0.97		0.88	-	1.07	0.99		0.91	-	1.08
Generation 1.5	1.07		0.93	-	1.23	0.99		0.86	-	1.13
Generation 2	0.90	*	0.81	-	1.00	0.97		0.89	-	1.06
Circulatory diseases										
Ancestral Swedes	0.79	**	0.75	-	0.83	0.26	**	0.24	-	0.28
Generation 1	0.77	**	0.69	-	0.86	0.21	**	0.18	-	0.25
Generation 1.5	0.84	*	0.72	-	0.99	0.25	**	0.19	-	0.32
Generation 2	0.84	**	0.75	-	0.93	0.26	**	0.22	-	0.31
Other diseases										
Ancestral Swedes	1.03		0.98	-	1.08	0.51	**	0.48	-	0.53
Generation 1	0.59	**	0.52	-	0.67	0.33	**	0.29	-	0.38
Generation 1.5	1.17	*	1.02	-	1.34	0.57	**	0.48	-	0.68
Generation 2	1.21	**	1.11	-	1.33	0.59	**	0.53	-	0.66
Accidents & injuries										
Ancestral Swedes	1.27	**	1.21	-	1.34	0.28	**	0.26	-	0.30
Generation 1	0.71	**	0.63	-	0.80	0.14	**	0.11	-	0.17
Generation 1.5	1.57	**	1.39	-	1.77	0.31	**	0.25	-	0.39
Generation 2	1.51	**	1.39	-	1.63	0.35	**	0.30	-	0.40
Suicides										
Ancestral Swedes	1.51	**	1.44	-	1.58	0.47	**	0.45	-	0.50
Generation 1	0.83	**	0.74	-	0.92	0.28	**	0.24	-	0.33
Generation 1.5	2.04	**	1.83	-	2.26	0.84	*	0.73	-	0.98
Generation 2	2.10	**	1.95	-	2.25	0.76	**	0.69	-	0.84
Substance Use										
Ancestral Swedes	0.95	*	0.90	-	1.00	0.18	**	0.16	-	0.19
Generation 1	0.37	**	0.31	-	0.43	0.07	**	0.05	-	0.10
Generation 1.5	1.66	**	1.48	-	1.87	0.22	**	0.17	-	0.29
Generation 2	1.76	**	1.63	-	1.91	0.32	**	0.28	-	0.38
Other external causes										
Ancestral Swedes	0.24	**	0.22	-	0.26	0.07	**	0.06	-	0.08
Generation 1	0.36	**	0.30	-	0.42	0.14	**	0.11	-	0.17
Generation 1.5	1.05		0.91	-	1.21	0.15	**	0.11	-	0.21
Generation 2	0.52	**	0.46	-	0.60	0.11	**	0.09	-	0.14
Ill-defined										
Ancestral Swedes	0.21	**	0.19	-	0.23	0.07	**	0.06	-	0.08
Generation 1	0.70	**	0.63	-	0.79	0.22	**	0.19	-	0.26
Generation 1.5	0.57	**	0.47	-	0.69	0.17	**	0.13	-	0.24
Generation 2	0.42	**	0.37	-	0.49	0.16	**	0.13	-	0.19
**Birth year**	0.99	**	0.98	-	0.99	0.98	**	0.98	-	0.99

Notes: p<0.01 **; p<0.05 *; p<0.1 +

Source: author's calculation based upon the Swedish register data collection "Ageing Well"

Table S7. Model 2b, extended, competing-risks survival model, men aged 15-44, 1997-2016, generation by western, non-western origins, cancer among ancestral Swedes as reference.

**Model 2b, men**	HR	Sig.	95%CIs				HR	Sig.	95%CIs		
**Cancers**						**Suicides**					
Ancestral Swedes	**1**					Ancestral Swedes	1.51	**	1.44	-	1.58
G1 Western	1.01		0.87	-	1.16	G1 Western	1.03		0.89	-	1.18
G1 Non-Western	0.94		0.82	-	1.07	G1 Non-Western	0.67	**	0.58	-	0.78
G1.5 Western	1.23	*	1.02	-	1.47	G1.5 Western	1.94	**	1.68	-	2.25
G1.5 Non-Western	0.89		0.72	-	1.11	G1.5 Non-Western	2.13	**	1.84	-	2.46
G2 Western	0.95		0.85	-	1.05	G2 Western	2.14	**	1.98	-	2.31
G2 Non-Western	0.58	**	0.42	-	0.80	G2 Non-Western	1.82	**	1.51	-	2.19
**Circulatory diseases**						**Substance Use**					
Ancestral Swedes	0.79	**	0.75	-	0.83	Ancestral Swedes	0.95	*	0.90	-	1.00
G1 Western	0.90		0.78	-	1.05	G1 Western	0.40	**	0.32	-	0.49
G1 Non-Western	0.66	**	0.57	-	0.77	G1 Non-Western	0.34	**	0.28	-	0.42
G1.5 Western	1.12		0.92	-	1.35	G1.5 Western	1.73	**	1.48	-	2.01
G1.5 Non-Western	0.55	**	0.42	-	0.72	G1.5 Non-Western	1.59	**	1.35	-	1.88
G2 Western	0.90	+	0.81	-	1.00	G2 Western	1.79	**	1.65	-	1.94
G2 Non-Western	0.44	**	0.30	-	0.64	G2 Non-Western	1.58	**	1.30	-	1.93
**Other diseases & medical conditions**						**Other external causes**					
Ancestral Swedes	1.03		0.98	-	1.08	Ancestral Swedes	0.24	**	0.22	-	0.26
G1 Western	0.69	**	0.58	-	0.82	G1 Western	0.33	**	0.26	-	0.42
G1 Non-Western	0.51	**	0.43	-	0.61	G1 Non-Western	0.38	**	0.31	-	0.46
G1.5 Western	1.40	**	1.18	-	1.66	G1.5 Western	0.73	**	0.58	-	0.92
G1.5 Non-Western	0.91		0.74	-	1.13	G1.5 Non-Western	1.39	**	1.16	-	1.66
G2 Western	1.26	**	1.14	-	1.38	G2 Western	0.44	**	0.38	-	0.51
G2 Non-Western	0.92		0.71	-	1.20	G2 Non-Western	1.06		0.84	-	1.35
**Accidents & injuries**						**Ill-defined**					
Ancestral Swedes	1.27	**	1.21	-	1.34	Ancestral Swedes	0.21	**	0.19	-	0.23
G1 Western	0.84	*	0.72	-	0.98	G1 Western	0.87	+	0.75	-	1.01
G1 Non-Western	0.61	**	0.52	-	0.72	G1 Non-Western	0.57	**	0.48	-	0.67
G1.5 Western	1.64	**	1.40	-	1.93	G1.5 Western	0.66	**	0.52	-	0.84
G1.5 Non-Western	1.48	**	1.25	-	1.76	G1.5 Non-Western	0.47	**	0.35	-	0.64
G2 Western	1.52	**	1.40	-	1.66	G2 Western	0.38	**	0.33	-	0.45
G2 Non-Western	1.38	**	1.11	-	1.70	G2 Non-Western	0.67	**	0.50	-	0.91

Notes: p<0.01 **; p<0.05 *; p<0.1 +

Source: author's calculation based upon the Swedish register data collection "Ageing Well"

Table S8. Model 2b, extended, competing-risks survival model, women aged 15-44, 1997-2016, generation by western, non-western origins, cancer among ancestral Swedes as reference.

**Model 2b, women**	HR	Sig.	95%CIs				HR	Sig.	95%CIs		
**Cancers**						**Suicides**					
Ancestral Swedes	**1**					Ancestral Swedes	0.47	**	0.45	-	0.50
G1 Western	1.04		0.93	-	1.17	G1 Western	0.36	**	0.30	-	0.43
G1 Non-Western	0.96		0.85	-	1.07	G1 Non-Western	0.21	**	0.17	-	0.27
G1.5 Western	1.15		0.97	-	1.36	G1.5 Western	0.75	**	0.61	-	0.92
G1.5 Non-Western	0.81	+	0.66	-	1.00	G1.5 Non-Western	0.95		0.78	-	1.15
G2 Western	1.01		0.92	-	1.11	G2 Western	0.76	**	0.68	-	0.84
G2 Non-Western	0.66	**	0.49	-	0.88	G2 Non-Western	0.77	+	0.59	-	1.02
**Circulatory diseases**						**Substance Use**					
Ancestral Swedes	0.26	**	0.24	-	0.28	Ancestral Swedes	0.18	**	0.16	-	0.19
G1 Western	0.23	**	0.18	-	0.29	G1 Western	0.11	**	0.08	-	0.15
G1 Non-Western	0.20	**	0.16	-	0.25	G1 Non-Western	0.04	**	0.03	-	0.07
G1.5 Western	0.33	**	0.24	-	0.45	G1.5 Western	0.22	**	0.15	-	0.32
G1.5 Non-Western	0.16	**	0.10	-	0.26	G1.5 Non-Western	0.23	**	0.15	-	0.34
G2 Western	0.27	**	0.23	-	0.32	G2 Western	0.32	**	0.27	-	0.38
G2 Non-Western	0.16	**	0.09	-	0.29	G2 Non-Western	0.34	**	0.22	-	0.51
**Other diseases & medical conditions**						**Other external causes**					
Ancestral Swedes	0.51	**	0.48	-	0.53	Ancestral Swedes	0.07	**	0.06	-	0.08
G1 Western	0.29	**	0.24	-	0.36	G1 Western	0.12	**	0.08	-	0.16
G1 Non-Western	0.36	**	0.30	-	0.43	G1 Non-Western	0.16	**	0.12	-	0.21
G1.5 Western	0.60	**	0.47	-	0.75	G1.5 Western	0.12	**	0.07	-	0.20
G1.5 Non-Western	0.54	**	0.42	-	0.70	G1.5 Non-Western	0.18	**	0.12	-	0.28
G2 Western	0.58	**	0.52	-	0.66	G2 Western	0.11	**	0.08	-	0.14
G2 Non-Western	0.64	**	0.48	-	0.87	G2 Non-Western	0.10	**	0.05	-	0.21
**Accidents & injuries**						**Ill-defined**					
Ancestral Swedes	0.28	**	0.26	-	0.30	Ancestral Swedes	0.07	**	0.06	-	0.08
G1 Western	0.16	**	0.12	-	0.21	G1 Western	0.24	**	0.19	-	0.30
G1 Non-Western	0.12	**	0.09	-	0.17	G1 Non-Western	0.21	**	0.17	-	0.27
G1.5 Western	0.30	**	0.22	-	0.42	G1.5 Western	0.16	**	0.10	-	0.25
G1.5 Non-Western	0.32	**	0.23	-	0.44	G1.5 Non-Western	0.19	**	0.12	-	0.29
G2 Western	0.34	**	0.29	-	0.39	G2 Western	0.15	**	0.12	-	0.18
G2 Non-Western	0.42	**	0.29	-	0.61	G2 Non-Western	0.22	**	0.13	-	0.36

Notes: $p<0.01$ **; $p<0.05$ *; $p<0.1$ +

Source: author's calculation based upon the Swedish register data collection "Ageing Well"

Table S9. Model 2c, extended, competing-risks survival model, men aged 15-44, 1997-2016, generation by lowest level origins, cancer among ancestral Swedes as reference.

Model 2c, men	Cancers			Circulatory			Other diseases			Accidents			Suicides			Substance Use			Other external			Ill-defined		
	HR	95%CIs	Sig.	HR	95%CIs	Sig.	HR	95%CIs	Sig.	HR	95%CIs	Sig.	HR	95%CIs	Sig.	HR	95%CIs	Sig.	HR	95%CIs	Sig.	HR	95%CIs	Sig.
Ancestral Swedes	1			0.79	0.75 - 0.83	**	1.03	0.98 - 1.08		1.27	1.21 - 1.34	**	1.51	1.44 - 1.58	**	0.95	0.90 - 1.00	*	0.24	0.22 - 0.26	**	0.21	0.19 - 0.23	**
Generation 1																								
Finland	1.43	0.96 - 2.11	+	2.97	2.25 - 3.90	**	2.97	2.25 - 3.90	**	2.17	1.57 - 2.99	**	2.62	1.96 - 3.51	**	1.03	0.65 - 1.63		0.68	0.39 - 1.21		1.65	1.15 - 2.39	**
Other Nordic	0.81	0.52 - 1.26		0.61	0.37 - 1.01	+	0.40	0.22 - 0.75	**	0.85	0.55 - 1.31		1.33	0.95 - 1.88	+	0.40	0.22 - 0.75	**	0.16	0.06 - 0.43	**	1.09	0.75 - 1.60	
Other Western	0.76	0.57 - 1.02	+	0.46	0.32 - 0.67	**	0.40	0.27 - 0.59	**	0.45	0.31 - 0.65	**	0.76	0.57 - 1.02	+	0.28	0.17 - 0.45	**	0.10	0.04 - 0.22	**	0.61	0.44 - 0.85	**
Central Eastern Europe	1.13	0.94 - 1.36		0.89	0.72 - 1.09		0.55	0.42 - 0.71	**	0.84	0.68 - 1.04		0.84	0.68 - 1.04		0.36	0.26 - 0.49	**	0.44	0.33 - 0.59	**	0.84	0.68 - 1.04	
The Middle East	1.00	0.84 - 1.18		0.72	0.60 - 0.88	**	0.40	0.30 - 0.51	**	0.61	0.49 - 0.75	**	0.61	0.50 - 0.76	**	0.44	0.35 - 0.57	**	0.37	0.28 - 0.48	**	0.48	0.38 - 0.61	**
Central Southern America	0.83	0.53 - 1.29		0.46	0.25 - 0.83	**	0.41	0.22 - 0.77	**	0.50	0.28 - 0.88	*	1.12	0.77 - 1.64		0.37	0.19 - 0.72	**	0.25	0.11 - 0.55	**	0.62	0.37 - 1.03	+
Sub-Saharan Africa	1.12	0.85 - 1.48		0.84	0.61 - 1.15		1.12	0.85 - 1.48		0.82	0.59 - 1.13		0.97	0.72 - 1.31		0.24	0.13 - 0.44	**	0.57	0.39 - 0.84	**	0.97	0.72 - 1.31	
Asia	0.61	0.42 - 0.89	**	0.41	0.26 - 0.65	**	0.33	0.20 - 0.54	**	0.48	0.32 - 0.73	**	0.33	0.20 - 0.54	**	0.11	0.05 - 0.26	**	0.28	0.16 - 0.49	**	0.41	0.26 - 0.65	**
Generation 1.5																								
Finland	1.78	1.36 - 2.33	**	1.91	1.47 - 2.48	**	2.47	1.97 - 3.11	**	2.31	1.82 - 2.93	**	3.00	2.43 - 3.70	**	2.04	1.59 - 2.63	**	1.22	0.88 - 1.69		0.53	0.32 - 0.86	*
Other Nordic	0.43	0.18 - 1.02	+	1.11	0.64 - 1.91		1.45	0.90 - 2.33		1.19	0.70 - 2.01		1.45	0.90 - 2.33		1.53	0.96 - 2.43	+	0.17	0.04 - 0.68	*	0.51	0.23 - 1.14	
Other Western	1.40	0.93 - 2.11		1.04	0.64 - 1.67		0.85	0.50 - 1.44		0.85	0.50 - 1.44		1.46	0.98 - 2.19	+	1.40	0.93 - 2.11		0.24	0.09 - 0.65	**	0.79	0.46 - 1.37	
Central Eastern Europe	0.97	0.71 - 1.34		0.55	0.36 - 0.84	**	0.80	0.56 - 1.13		1.60	1.25 - 2.05	**	1.47	1.14 - 1.91	**	1.67	1.31 - 2.13	**	0.72	0.50 - 1.04	+	0.75	0.52 - 1.07	
The Middle East	0.91	0.65 - 1.26		0.38	0.23 - 0.63	**	0.61	0.40 - 0.90	**	1.44	1.11 - 1.87	**	1.34	1.02 - 1.75	*	1.74	1.37 - 2.21	**	1.77	1.39 - 2.24	**	0.50	0.32 - 0.78	**
Central Southern America	0.84	0.52 - 1.35		0.64	0.37 - 1.10		1.13	0.75 - 1.70		1.33	0.91 - 1.94		2.36	1.77 - 3.14	**	1.72	1.23 - 2.40	**	0.84	0.52 - 1.35		0.25	0.10 - 0.59	**
Sub-Saharan Africa	0.79	0.39 - 1.57		0.30	0.10 - 0.92	*	1.87	1.19 - 2.93	**	1.67	1.04 - 2.69	*	2.26	1.50 - 3.41	**	1.77	1.11 - 2.81	*	2.85	1.98 - 4.11	**	0.89	0.46 - 1.70	
Asia	0.94	0.62 - 1.43		0.85	0.55 - 1.33		0.81	0.52 - 1.27		1.58	1.14 - 2.19	**	3.16	2.51 - 3.98	**	1.11	0.75 - 1.63		0.56	0.32 - 0.96	*	0.43	0.23 - 0.79	**
Generation 2																								
Finland	0.98	0.84 - 1.14		1.06	0.91 - 1.22		1.52	1.34 - 1.72	**	1.83	1.63 - 2.05	**	2.59	2.35 - 2.86	**	2.27	2.04 - 2.52	**	0.50	0.40 - 0.62	**	0.39	0.31 - 0.49	**
Other Nordic	0.86	0.66 - 1.11		0.80	0.61 - 1.04	+	1.39	1.14 - 1.71	**	1.48	1.21 - 1.80	**	2.10	1.78 - 2.49	**	1.36	1.11 - 1.67	**	0.32	0.21 - 0.49	**	0.19	0.11 - 0.33	**
Other Western	1.07	0.87 - 1.31		0.78	0.62 - 0.99	*	0.85	0.68 - 1.06		1.22	1.01 - 1.48	*	1.61	1.36 - 1.90	**	1.14	0.93 - 1.38		0.39	0.28 - 0.54	**	0.50	0.37 - 0.67	**
Central Eastern Europe	0.80	0.62 - 1.04	+	0.76	0.58 - 0.99	*	0.99	0.79 - 1.25		1.19	0.96 - 1.47		1.71	1.43 - 2.05	**	1.85	1.55 - 2.20	**	0.47	0.33 - 0.66	**	0.41	0.29 - 0.59	**
The Middle East	0.60	0.39 - 0.94	*	0.48	0.30 - 0.79	**	0.97	0.68 - 1.37		1.33	0.99 - 1.79	+	1.45	1.09 - 1.93	*	1.57	1.19 - 2.07	**	1.33	0.99 - 1.79	+	0.57	0.37 - 0.90	*
Central Southern America	0.57	0.27 - 1.21		0.33	0.12 - 0.87	*	0.98	0.56 - 1.74		1.56	0.99 - 2.45	+	1.97	1.32 - 2.94	**	1.97	1.32 - 2.94	**	0.66	0.33 - 1.31		0.57	0.27 - 1.21	
Sub-Saharan Africa	0.45	0.14 - 1.39		0.60	0.22 - 1.59		1.19	0.59 - 2.38		1.64	0.91 - 2.96		2.83	1.80 - 4.44	**	1.04	0.50 - 2.19		1.79	1.01 - 3.15	*	1.19	0.59 - 2.38	
Asia	0.56	0.27 - 1.18		0.32	0.12 - 0.85	*	0.56	0.27 - 1.18		1.12	0.66 - 1.89		2.00	1.35 - 2.96	**	1.44	0.91 - 2.29		0.32	0.12 - 0.85	*	0.72	0.37 - 1.38	

Notes: p<0.01 **; p<0.05 *; p<0.1 +

Source: author’s calculation based upon the Swedish register data collection “Ageing Well”

Table S10. Model 2c, extended, competing-risks survival model, women aged 15-44, 1997-2016, , generation by lowest level origins, cancer among ancestral Swedes as reference..

**Model 2c, women**	**Cancers**			**Circulatory**			**Other diseases**			**Accidents**			**Suicides**			**Substance Use**			**Other external**			**Ill-defined**		
	HR	95%CIs	Sig.	HR	95%CIs	Sig.	HR	95%CIs	Sig.	HR	95%CIs	Sig.	HR	95%CIs	Sig.	HR	95%CIs	Sig.	HR	95%CIs	Sig.	HR	95%CIs	Sig.
Ancestral Swedes	**1**			0.26	0.24 - 0.28	**	0.51	0.48 - 0.53	**	0.28	0.26 - 0.30	**	0.47	0.45 - 0.50	**	0.18	0.16 - 0.19	**	0.07	0.06 - 0.08	**	0.07	0.06 - 0.08	**
Generation 1																								
Finland	1.38	1.06 - 1.80	*	0.50	0.32 - 0.78	**	0.65	0.44 - 0.96	*	0.38	0.23 - 0.63	**	0.68	0.46 - 0.99	*	0.25	0.14 - 0.47	**	0.13	0.05 - 0.30	**	0.18	0.08 - 0.37	**
Other Nordic	0.84	0.56 - 1.24		0.30	0.16 - 0.58	**	0.33	0.18 - 0.62	**	0.17	0.07 - 0.40	**	0.43	0.25 - 0.75	**	0.13	0.05 - 0.36	**	0.03	0.00 - 0.24	**	0.17	0.07 - 0.40	**
Other Western	0.68	0.49 - 0.94	*	0.13	0.06 - 0.28	**	0.23	0.13 - 0.40	**	0.02	0.00 - 0.13	**	0.23	0.13 - 0.40	**	0.08	0.03 - 0.20	**	0.06	0.02 - 0.18	**	0.32	0.20 - 0.52	**
Central Eastern Europe	1.11	0.96 - 1.28		0.18	0.13 - 0.25	**	0.22	0.16 - 0.31	**	0.15	0.10 - 0.22	**	0.31	0.24 - 0.41	**	0.08	0.05 - 0.13	**	0.15	0.10 - 0.22	**	0.24	0.18 - 0.33	**
The Middle East	0.94	0.80 - 1.10		0.14	0.09 - 0.21	**	0.17	0.11 - 0.24	**	0.10	0.06 - 0.16	**	0.12	0.07 - 0.18	**	0.02	0.01 - 0.07	**	0.18	0.12 - 0.26	**	0.16	0.11 - 0.24	**
Central Southern America	0.88	0.63 - 1.24		0.21	0.11 - 0.43	**	0.24	0.13 - 0.46	**	0.05	0.01 - 0.21	**	0.32	0.18 - 0.57	**	0.08	0.03 - 0.25	**	0.05	0.01 - 0.21	**	0.19	0.09 - 0.39	**
Sub-Saharan Africa	1.21	0.95 - 1.54		0.37	0.24 - 0.58	**	1.07	0.83 - 1.38		0.20	0.11 - 0.35	**	0.18	0.10 - 0.33	**	0.07	0.03 - 0.19	**	0.23	0.13 - 0.40	**	0.32	0.20 - 0.51	**
Asia	0.87	0.70 - 1.08		0.19	0.12 - 0.30	**	0.31	0.22 - 0.45	**	0.15	0.09 - 0.25	**	0.35	0.25 - 0.49	**	0.04	0.02 - 0.11	**	0.13	0.08 - 0.23	**	0.24	0.16 - 0.36	**
Generation 1.5																								
Finland	1.45	1.12 - 1.87	**	0.50	0.32 - 0.77	**	0.59	0.40 - 0.88	**	0.28	0.16 - 0.50	**	1.00	0.74 - 1.35		0.31	0.18 - 0.53	**	0.07	0.02 - 0.22	**	0.09	0.04 - 0.25	**
Other Nordic	1.43	0.93 - 2.19		0.34	0.14 - 0.82	*	0.75	0.41 - 1.35		0.20	0.07 - 0.63	**	0.61	0.32 - 1.18		0.20	0.07 - 0.63	**	0.27	0.10 - 0.73	**	0.27	0.10 - 0.73	**
Other Western	0.93	0.59 - 1.49		0.05	0.01 - 0.37	**	0.78	0.47 - 1.29		0.36	0.17 - 0.76	**	0.26	0.11 - 0.62	**	0.21	0.08 - 0.55	**	0.00	0.00 - 0.00		0.21	0.08 - 0.55	**
Central Eastern Europe	0.86	0.62 - 1.19		0.28	0.16 - 0.49	**	0.46	0.30 - 0.72	**	0.33	0.19 - 0.55	**	0.77	0.54 - 1.08		0.14	0.06 - 0.31	**	0.16	0.08 - 0.34	**	0.16	0.08 - 0.34	**
The Middle East	0.66	0.45 - 0.97	*	0.05	0.01 - 0.20	**	0.48	0.31 - 0.75	**	0.30	0.17 - 0.53	**	0.56	0.37 - 0.85	**	0.23	0.12 - 0.44	**	0.23	0.12 - 0.44	**	0.15	0.07 - 0.34	**
Central Southern America	0.80	0.50 - 1.28		0.14	0.05 - 0.44	**	0.38	0.19 - 0.75	**	0.42	0.22 - 0.81	**	0.61	0.35 - 1.05	+	0.19	0.07 - 0.50	**	0.19	0.07 - 0.50	**	0.23	0.10 - 0.56	**
Sub-Saharan Africa	1.12	0.64 - 1.97		0.65	0.31 - 1.37		0.84	0.44 - 1.62		0.47	0.19 - 1.12	+	1.31	0.77 - 2.21		0.19	0.05 - 0.75	*	0.09	0.01 - 0.66	*	0.56	0.25 - 1.25	
Asia	0.88	0.63 - 1.24		0.16	0.07 - 0.35	**	0.60	0.40 - 0.90	**	0.23	0.12 - 0.45	**	1.43	1.10 - 1.87	**	0.26	0.14 - 0.48	**	0.16	0.07 - 0.35	**	0.10	0.04 - 0.28	**
Generation 2																								
Finland	0.97	0.85 - 1.12		0.31	0.25 - 0.40	**	0.58	0.48 - 0.69	**	0.38	0.30 - 0.47	**	0.97	0.84 - 1.11		0.43	0.35 - 0.53	**	0.12	0.08 - 0.17	**	0.15	0.10 - 0.21	**
Other Nordic	1.15	0.94 - 1.40		0.29	0.20 - 0.43	**	0.68	0.52 - 0.88	**	0.33	0.23 - 0.48	**	0.66	0.50 - 0.85	**	0.23	0.15 - 0.36	**	0.12	0.06 - 0.22	**	0.11	0.05 - 0.20	**
Other Western	0.95	0.78 - 1.15		0.23	0.15 - 0.34	**	0.53	0.41 - 0.69	**	0.29	0.21 - 0.41	**	0.48	0.37 - 0.63	**	0.21	0.14 - 0.32	**	0.08	0.04 - 0.16	**	0.12	0.07 - 0.20	**
Central Eastern Europe	1.06	0.86 - 1.31		0.21	0.13 - 0.33	**	0.59	0.44 - 0.77	**	0.30	0.21 - 0.45	**	0.67	0.51 - 0.87	**	0.27	0.18 - 0.41	**	0.13	0.07 - 0.23	**	0.22	0.14 - 0.35	**
The Middle East	0.68	0.46 - 1.02	+	0.17	0.08 - 0.38	**	0.51	0.32 - 0.82	**	0.66	0.43 - 0.99	*	0.40	0.24 - 0.67	**	0.40	0.24 - 0.67	**	0.11	0.04 - 0.30	**	0.29	0.15 - 0.53	**
Central Southern America	0.47	0.21 - 1.05	+	0.00	0.00 - 0.00		0.86	0.48 - 1.56		0.08	0.01 - 0.56	**	0.94	0.53 - 1.65		0.23	0.08 - 0.73	**	0.08	0.01 - 0.56	*	0.08	0.01 - 0.56	*
Sub-Saharan Africa	0.26	0.07 - 1.05	+	0.53	0.20 - 1.40		0.66	0.27 - 1.58		0.39	0.13 - 1.22		1.31	0.71 - 2.45		0.13	0.02 - 0.93	*	0.13	0.02 - 0.93	*	0.26	0.07 - 1.05	+
Asia	0.98	0.57 - 1.69		0.08	0.01 - 0.54	**	0.75	0.40 - 1.40		0.15	0.04 - 0.60	**	1.28	0.80 - 2.06		0.38	0.16 - 0.91	*	0.08	0.01 - 0.54	**	0.15	0.04 - 0.60	**

Notes: p<0.01 **; p<0.05 *; p<0.1 +

Source: author's calculation based upon the Swedish register data collection "Ageing Well"

Contributions of specific causes of death to overall mortality

The contributions of specific causes to the observed all-cause mortality differentials between migrant-origin populations (i.e., the **G1**, **G15**, and **G2**) can be calculated from Models 2a-c as follows:

$$hr\_c_{zk} = \left(\frac{hr_{zk}(t)}{\sum hr_{zk}(t)} \times \frac{\sum hr_{zk}(t)}{\sum hr_{Zk}(t)}\right) - \left(\frac{hr_{Zk}(t)}{\sum hr_{Zk}(t)}\right)$$

Whereby a lowercase **z** refers to specific groups of migrants and their descendants, while an uppercase $\boldsymbol{Z}$ instead refers to the reference group i.e., the ancestral Swedes. First, divide the hazard ratio **for cause** $\boldsymbol{k}$ for migrant origin group **z** (i.e.., $\boldsymbol{hr_{zk}(t)}$) by the sum of the $\boldsymbol{k}$ cause-specific hazard ratios for migrant-origin group $\boldsymbol{z}$ (i.e., $\sum \boldsymbol{hr_{zk}(t)}$). *NB: the effect of the covariates is additive on this scale.* Next, multiply this value by the value of the sum of the $\boldsymbol{k}$ cause-specific hazard ratios for migrant-origin group $\boldsymbol{z}$ divided by the sum of the $\boldsymbol{k}$ cause-specific hazard ratios for ancestral Swedes (i.e., $\sum \boldsymbol{hr_{Zk}(t)}$). Finally, from this value, subtract the value of the hazard ratio for specific cause $\boldsymbol{k}$ for the ancestral Swedish group (i.e.., $\boldsymbol{hr_{Zk}(t)}$) divided by the sum of all of the $\boldsymbol{k}$ cause-specific hazard ratios for the ancestral Swedes (i.e., $\sum \boldsymbol{hr_{Zk}(t)}$).

Note that $\frac{\sum \boldsymbol{hr_{zk}(t)}}{\sum \boldsymbol{hr_{Zk}(t)}}$ (i.e., the sum of cause-specific hazard ratios from Models 2a-c for migrant-origin group $\boldsymbol{z}$ divided by the sum of the cause-specific hazard ratios for the ancestral Swedes $\boldsymbol{Z}$) generates the all-cause hazard ratio from Models 1a-c for the group for which the calculation is made.

Finally, note that the sum of the contributions of all specific causes of death for a given migrant-origin group **+ 1** gives the mortality hazard ratio from Models 1a-c for the group for which the calculation is made. One could also replace **1** with $\boldsymbol{hr_{Z}(t)}$ (i.e., the reference for the ancestral Swedes).

$$hr_z(t) = 1 + \sum hr\_c_{zk}$$

Below, an example is provided from Model 2a for men belonging to the **G15**. We can follow with Table 1.

Table 1. Calculating hazard ratio contributions for the G15, men

Generation	Cause of death	(a)	(b)	(c)	(d)
Ancestral Swedes (AS)	Cancer	1.00	0.14	-	-
	Circulatory	0.79	0.11	-	-
	Other diseases	1.14	0.16	-	-
	Accidents	1.81	0.26	-	-
	Suicides	1.50	0.21	-	-
	Other external	0.55	0.08	-	-
	Ill-defined	0.19	0.03		-
**Sum of HRs**		**6.97**		**1.00**	**-**
G15	Cancer	1.07	0.11	0.15	0.01
	Circulatory	0.85	0.08	0.12	0.01
	Other diseases	1.52	0.15	0.22	0.05
	Accidents	2.69	0.27	0.39	0.13
	Suicides	2.06	0.20	0.30	0.08
	Other external	1.45	0.14	0.21	0.13
	Ill-defined	0.47	0.05	0.07	0.04
**Sum of HRs**		**10.10**		**1.45**	**0.45**
**Ratio vs. ancestral Swedes**		**1.45**		**1.45**	**-**

$$hr\_c_{G15\_accidents} = \left(\frac{2.69}{10.10} \times \frac{10.10}{6.97}\right) - \left(\frac{1.81}{6.97}\right) = +0.13$$

The values in column a are taken directly from Model 2a. Let us calculate the contribution for accident mortality. **G15** men have a hazard ratio from accident mortality of 2.69 in Model 2a. We first divide 2.69 by the sum of all of the cause-specific mortality hazard ratios for **G15** men (10.10) to give a value of 0.27 (column b). Next, we multiply this value of 0.27 by 1.45 (i.e., the sum of all of the cause-specific **G15** hazard ratios [10.10] divided by the sum of all of the cause-specific hazard ratios for ancestral Swedish men [6.97]) to give a value of 0.39 (column c). One could alternatively, just use the all-cause hazard ratio for the **G15** from Model 1a.

Finally, from this value of 0.39 we can subtract the value for accidents in ancestral Swedes (0.26) to give a +0.13 contribution (column d) of accidents among **G15** men to their all-cause hazard ratio vs. ancestral Swedes. 1 + the sum of the respective contributions of each cause, as column d shows would then equates to the all-cause mortality hazard ratio from Model 1a (HR 1.45).
